# Proteomic analysis of embryonic kidney development: Heterochromatin proteins as epigenetic regulators of nephrogenesis

**DOI:** 10.1038/srep13951

**Published:** 2015-09-11

**Authors:** Gry H. Dihazi, Olaf Jahn, Björn Tampe, Michael Zeisberg, Claudia Müller, Gerhard A. Müller, Hassan Dihazi

**Affiliations:** 1 Department of Nephrology and Rheumatology, Georg-August University Göttingen, Robert-Koch-Strasse 40, D-37075 Göttingen, Germany; 2 Proteomics Group, Max-Planck-Institute of Experimental Medicine, Hermann-Rein-Strasse 3, D-37075 Göttingen, Germany; 3 Deutsche Forschungsgemeinschaft Research Center for Molecular Physiology of the Brain, Humboldtallee 23, D-37073 Göttingen, Germany; 4 Section for Transplantation- Immunology and Immunohematology, ZMF, Eberhard-Karls-University Tübingen, Germany

## Abstract

Elucidation of the mechanisms underlying the nephrogenesis will boost enormously the regenerative medicine. Here we performed 2-D gel-based comparative proteome analyses of rat embryonic kidney from different developmental stages. Out of 288 non-redundant identified proteins, 102 were common in all developmental stages. 86% of the proteins found in E14 and E16 were identical, in contrast only 37% of the identified proteins overlap between E14 and P1. Bioinformatics analysis suggests developmental stage-specific pathway activation and highlighted heterochromatin protein 1 (Cbx1, Cbx3, Cbx5) and Trim28 as potential key players in nephrogenesis. These are involved in the epigenetic regulation of gene silencing and were down-regulated in the course of kidney development. Trim28 is a potential epigenetic regulator of the branching inhibitor Bmp4. Silencing of Trim28 in cultured kidneys resulted in branching arrest. In contrast knockdown of Cbx5 was associated with abnormal ureteric bud growth and slight impairment of branching. ChIP analysis showed that the H3K9me3 distribution on *Bmp4* promoters at E14 and E19 inversely correlate with mRNA expression levels. The concentrated expression-pattern of heterochromatin proteins and the negative impact of their silencing on kidney development, suggest an important role in reciprocal and inductive signaling between the ureteric bud and the metanephric mesenchyme.

During the nephrogenesis the kidney undergoes a succession of morphogenetic events, driven by cell growth and differentiation. The mechanisms governing the nephrogenesis have been intensively investigated in various animal models, such as fish^1^, amphibians^2^, and mice^3^, and some of the key proteins and pathways have been identified. Still the kidney development mechanisms remain less understood.

The embryonic kidney develops through three stages; the pronephros, and the mesonephros, which both will regress throughout development and the metanephros, which forms the final kidney^4^,^5^,^6^. The ureteric bud (UB) branches out of the caudal end of the Wollfian duct. Reciprocal induction between the UB and the metanephric mesenchyme (MM) results in developing nephron through mesenchymal-epithelial transition (MET), as well as in branching of the UB^7^. The UB branches will form the collecting system, including collecting duct, renal pelvis, ureter, and bladder trigone. The nephrons are induced at each ureteric tip and vascular development in the kidney occurs with the glomerular development^8^,^9^.

MET to form the nephron, and differentiation of highly specialized structures, like the glomerulus^10^, are processes, which are unique to the kidney, whereas epithelial-mesenchymal interactions, branching morphogenesis, stem and progenitor cell maintenance and differentiation, cell-migration oriented cell division, and cell-extracellular matrix interactions have common roles in other organs^11^. This makes the ability to perform organ culture, and then to manipulate and visualize early stages of the kidney development, attractive for studying the cellular and molecular basis of organogenesis^12^.

Relatively few molecular mechanisms are known to be capable of directing a diverse sequence of events during kidney organogenesis. Glia derived neurotrophic factor (Gdnf) signalling via its receptor Ret is the major signal for ureteric budding^13^. Gdnf, a secreted growth factor, is targeted by multiple transcription factors, and expressed in the MM. The effects of Gdnf are modulated by several other growth factors and inhibitors of bud branching. Ret, a proto-oncogene and tyrosine kinase receptor, is expressed in the Wollfian duct, with the highest expression level at the site of the UB outgrowth and later at the tips of the UB^14^,^15^. It seems to regulate sites of cell proliferation and branching. Retinoids are required for expression of Ret^16^, and their signalling stimulates cell proliferation and migration leading to invasion of the UB into the MM^17^,^18^. Apart from the Gdnf signalling, common molecular signalling by canonical Wnt/β-catenin^19^ and sonic hedgehog pathways^20^, as well as bone morphogenic proteins (BMP)^21^ and fibroblast growth factors (FGF)^22^,^23^ coordinate multiple aspects of renal development within both the MM and the UB.

In the present study we performed comparative proteomic analyses of embryonic kidney and highlighted the stage dependent activation of signalling pathways. Moreover, we identified the epigenetic regulator heterochromatin proteins as key players in nephrogenesis.

## Results

### Embryonic Kidney Preparation and HE Staining

**Figure 1:**
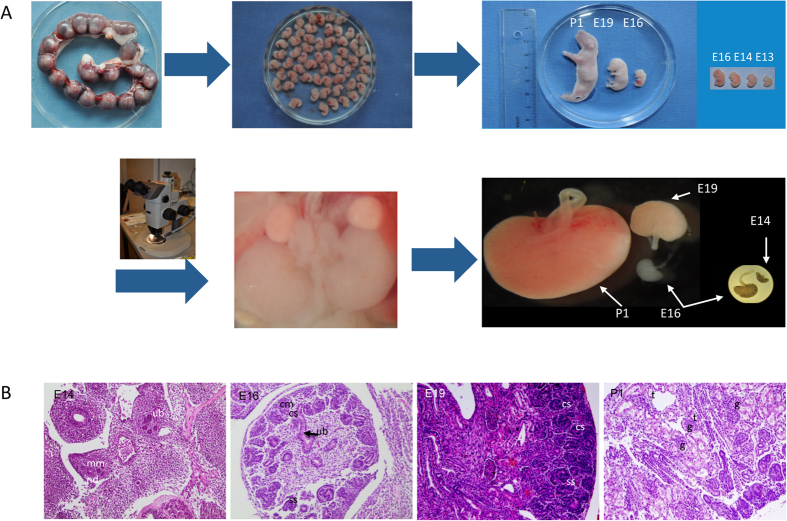
Embryonic kidney isolation. (**A**) Uterine horns containing the embryos were isolated from pregnant rats and the embryos were collected; Size comparison of embryos from different stages and from a newborn pup. Embryos were dissected under microscope and the kidneys were isolated; size comparison of the kidneys from different developmental stages. (**B**) HE staining of embryonic sections from different investigated stages, showing the metanephros including the outgrowth of the ureteric bud (ub), the nephric duct (nd), and the early formation of the metanephric mesenchyme (mm), comma-shaped (cs), s-shaped body (ss), cap mesenchyme (cm), glomerulus (g) and tubules (t).

Rat embryos at three different stages of development (E14, E16, E19), and neonatal pups (P1) were sacrificed and kidneys were obtained (Fig. 1A). Tissue section preparation and HE staining showed different developing stages of the kidney, which progress through complicated morphological structures as evidenced by HE staining of tissue sections from the different stages investigated (Fig. 1B). During the early stages of the kidney development the MM interacts with the nephric duct resulting in UB. By invading the MM the UB tips, each surrounded by a cap of MM (Fig. 1B), start a series of branching, which results in the metanephric collecting duct system^24^. Signals from the ureteric epithelial tip cells induce the MM to condense into cap-like structures. A part of these cells further aggregate to form pretubular aggregates, which undergo a MET giving rise to renal vesicles, which later differentiate into comma- and S-shaped bodies (Fig. 1B, E14 and E16). The upper limb of the S-shaped body fuses with the tip of the UB. The lower limb develops into glomerulus (podocytes and Bowman’s capsule) and the middle section differentiates and elongates into tubules (Fig. 1B, P1) forming the nephrons^11^. The HE staining confirmed that the embryonic stages investigated are ideal to explore the mechanism of kidney development on proteome level.

### Kidney Proteome Changes During Embryonic Development

**Figure 2:**
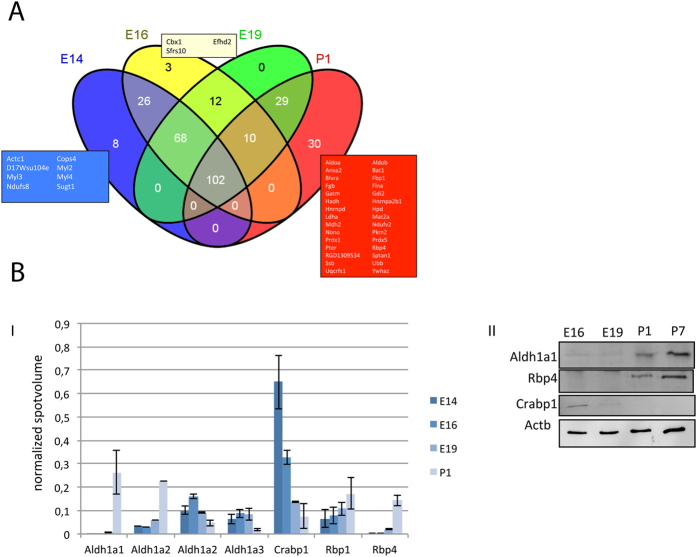
(**A**) Venn diagram of the identified proteins. 102 of the identified proteins were found in all developmental stages. Eight proteins were only present at E14, three proteins were unique to E16, and 30 proteins were only identified in P1. (**B**) Validation of proteomics data. I: Evaluation of the spot intensity of protein found to be involved in RA-pathway. The protein expression is regulated in the course of kidney development. II: Western blot analysis of some of the proteins, which are involved in the RA-pathway. Blots were performed with kidney extracts from E16 to P7 (seven days old pups) confirming the proteomics data. The full-length blotting images are presented in Supplemental Fig. 7.

To map the proteome changes underlying nephrogenesis, proteins were extracted from kidney homogenates and separated by 2-D gel electrophoresis (2-DE). By image analysis, around 1300 ± 150 protein spots were detected in the pH 5–8 region (Supplemental Fig. 1A–C). Comparison of the 2-DE maps from the four developmental stages, E14, E16, E19, and P1, revealed high similarity in the 2-DE pattern between E14 and E16, whereas E19 and P1 showed significant differences (Supplemental Fig. 1B). 977 protein spots were excised from the 2-DE gels, and processed for identification. A total of 685 protein spots were identified resulting in 288 non-redundant proteins (Supplemental Table 1). Comparative analysis of the protein expression, with regard to the embryonic stage, showed that 102 proteins were present in all four stages, whereas the other proteins were uniquely found in one, two or three stages (Fig. 2A)^25^. The highest similarities were observed between the proteomes at the stages E14 and E16, which had 86% of the identified proteins in common (Supplemental Fig. 2), eight proteins were expressed exclusively in E14 (Actc1, Cops4, D17Wsu104e, Myl2, Myl3, Myl4, Ndufs8, Sugt1) (Fig. 2A). Myl2 and Myl3 represent cardiac myosin forms, whereas Myl4 is an embryonic myosin form. 56% of the identified proteins overlapped between E19 and P1, whereas only 37% of the identified proteins overlapped between E14 and P1 (Supplemental Fig. 2). The retinoic acid pathway is an established pathway in kidney development and our proteomics data highlighted the regulation of several proteins from this pathway during the development. To validate the 2-DE data a selected group of the retinoic acid pathway proteins were tested for their expression during nephrogenesis using Western blot analyses (Fig. 2B). The Western blot data clearly confirmed the proteomics results for the selected proteins.

### Ontogenic Classification

**Figure 3:**
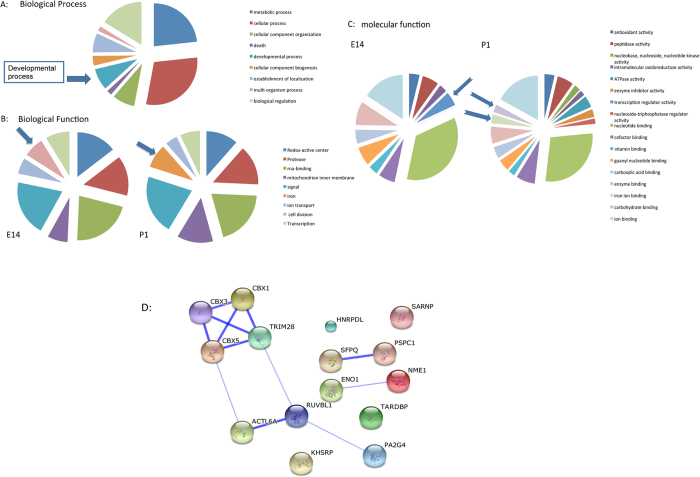
Classification of the identified proteins according to biological process. (**A**) Pie chart of the biological processes (GO annotations) in which the identified proteins are involved. Most of the proteins are involved in metabolic and cellular processes. A large part of the identified proteins were involved in developmental processes. (**B**) Pie charts of the classification of the identified proteins according to their biological functions. Proteins involved in cell division were identified at E14; these proteins were down-regulated in the course of development, whereas iron binding proteins were more prominent at P1. The other categories, redox-active center, protease, RNA-binding, mitochondrion inner membrane, signal, ion transport, and transcription were expressed in the different stages of kidney development. (**C**) Pie charts of the classification of the molecular function of the identified proteins. The classification revealed embryonic stage dependent expression of proteins involved in different pathways. Proteins involved in the category of transcription regulation decreased during the kidney development, other categories, like iron ion binding and carbohydrate binding were expressed in late stage of nephrogenesis. (**D**) Interaction map of proteins involved in transcription regulation. Strong interaction was observed between Trim28, Cbx1, Cbx3 and Cbx5.

To gain more information on the biological mechanisms associated with the embryonic development in the kidney of the identified proteins, we combined DAVID bioinformatics^26^,^27^ with information on the putative function of the proteins found in the UniProt and GenBank databases. The ontological classification of the proteins, which were identified at early stage of the kidney development, resulted in nine main categories (Fig. 3A). One of the mean categories was the developmental process, which includes subcategories like, organ development, embryonic development, embryonic organ development, kidney development, and mesenchyme development.

The identified proteins were also categorized according to their biological function (Fig. 3B). In E14 and P1 the kidney expresses mainly proteins involved in seven common categories. The category of cell division is only present at E14, whereas iron ion binding appears at P1 (Fig. 3B). In both stages, the most prominent categories were signal transduction and RNA-binding. An analysis of molecular function of the identified proteins in the kidney based on the Gene Ontology (GO) terms, allowed the differentiation of 17 different categories (Fig. 3C). Interestingly, when comparing the overall molecular function of identified and regulated proteins in the course of embryonic development, we showed that the group of proteins involved in transcription regulation and vitamin binding were significantly down-regulated from E14 to P1 (Fig. 3C), whereas iron ion binding and carbohydrate binding proteins, which are important for angiogenesis and vascularization, increased in course of development. Interestingly among the transcription factors, which were down-regulated in the course of kidney development, four heterochromatin proteins (Cbx1, Cbx3, Cbx5 and Trim28) attracted our attention because of their role in gene expression regulation and in stem cell pluripotency and differentiation.

### Protein-Protein Interaction Network in the Course of Kidney Development

Because protein-protein interactions are the key mechanism of almost all biological processes including development, extraction of information on possible protein-protein interaction and pathway regulation connecting the identified proteins could deliver significant information on nephrogenesis. To bring more light in the proteomic data and to get a general view on the protein functional interactions and to compare this in the course of kidney development we analysed the identified proteins using STRING 9.1 (http://string.embl.de/). The basic interaction of STRING is based on specific and meaningful interaction between proteins that contribute together to the same function. In our case, the networks generated showed strong association of some group of proteins revealing their interaction and importance during kidney embryonic development. For example, by analysing the proteins expressed at E14 and E16 the network showed an association of the down-regulated proteins, which were putatively associated with cardiac muscle contraction (Supplemental Fig. 3A,B). On the other hand the network revealed a strong interaction between proteins involved in retinoid metabolic process, which is known to play an important role in the kidney development (Supplemental Fig. 3C,D). When generating an interaction network with the down-regulated proteins involved in transcription regulation a strong association was achieved between four proteins Trim28, Cbx1, Cbx3, and Cbx5 (Fig. 3D). Trim28 contains an N-terminal RBCC (Ring finger, two B-box zinc fingers, and a coiled coil) domain, a central TIF1 signature sequence (TSS) domain and a HP1 (heterochromatin protein 1)-binding domain, which is a hydrophobic PxVxL pentapeptide, and a C terminal combination plant homeodomain (PHD) and bromodomain^28^. The RBCC domain of Trim28 interacts with a Krüppel associated box (KRAB)-domain transcription factor to increases the efficiency of KRAB-mediated repression. Cbx1, 3 and 5 belong to the family of heterochromatin protein 1 (HP1), which can bind to Trim28 at the PxVxL motif and also to H3K9me3, stabilizing the bound Trim28 complex , which is required for repression of reporter genes^29^.

To further examine the effect of proteins, which were regulated during the kidney development, a special focus was set on proteins, which expression decreased as the development progressed. Proteins, which are important in the transcription regulation activities, especially the family of the heterochromatin protein1 (HP1) (chromobox homolog, Cbx), and Trim28 attained a special focus. We could demonstrate in our former study, that these proteins, especially Trim28, are highly expressed in stem cells and play an important role in their differentiation^30^. Moreover an examination of the mRNA expression pattern of Trim28 as well as Cbx1, Cbx3 and Cbx5 in GUDMAP database^31^,^32^ (microarray expression analysis in mice embryonic kidney) confirmed the data obtained with proteomics in rat kidney. The data showed an overall expression of the genes in embryonic kidney E11.5–14.5 (rat E14-E17). The expression of the investigated genes decreased then after E15.5 (rat E18). These data corroborate with our results. In the rest of our work we aimed to explore the role of heterochromatin proteins in nephrogenesis.

### *Ex-vivo* Culture of Embryonic Kidney

**Figure 4:**
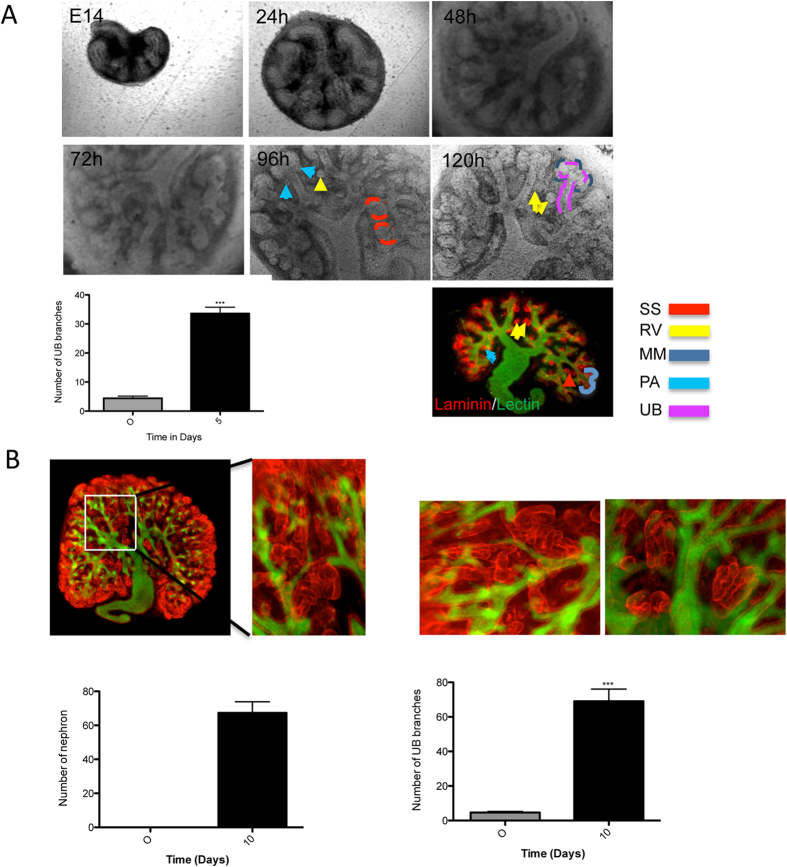
Temporal development of the cultured kidney. (**A**) The cultured kidney showed branching of the ureteric bud, condensation of the cap mesenchyme into pretubular aggregates, and development into renal vesicles, comma- and s-shaped bodies. After 5 days of culture, the kidneys were prepared for immunofluorescence staining. The figure shows a co-staining of laminin (red) and DBA lectin (green). The branching quantification was achieved by counting the number of branches of the ureteric bud. The quantification is presented as a bar chart with error bars. Each bar represents the branches number means ± s.d. of blots from 10 cultured rudiments. Significant differences: *p < 0.05, **p < 0.01, ***p < 0.001. (**B**) Kidney rudiment cultured for 10 days and stained with laminin to visualize the tubules (nephron) and with DBA lectin to visualize the ureteric bud. The kidney development was quantified by counting the number of branches and nephrons. The quantification is presented as a bar chart with error bars. Each bar represents the branches/nephron number means ± s.d. from 10 cultured rudiments. Significant differences: *p < 0.05, **p < 0.01, ***p < 0.001.

Organ culture, in particular kidney rudiment culture, provides a powerful tool to study renal development *ex-vivo*. This system has been proven to be useful for investigating the role of different proteins in kidney development using antibodies to inhibit protein function or siRNA to knockdown the protein of interest^33^,^34^. To investigate the role of the heterochromatin proteins, we established and optimized kidney^35^ rudiment culture (Supplemental Fig. 4). At the time of isolation the kidney rudiments had 2–6 UB tips. The cultured kidney developed normally, showing a well-branched UB (33.60 ± 2.135, n = 5) as well as different known structures from *in-vivo* developing kidney. The cultured kidney demonstrated pretubular aggregates, renal vesicles, S-shaped forms and cap mesenchyme (Fig. 4A) after four days of culture. Moreover, long-term culture resulted in a significant increase in the area of the organ rudiment, increase in number of UB tips (69.00 ± 7.109 N = 6), and increase in number of nephron (67.33 ± 6.546 N = 6). During this phase the renal vesicles elongate to form comma- and S-shaped bodies, the structure of which the glomerulus, Bowman’s capsule, proximal tubule, loop of Henle, and distal tubule are derived. To visualize the renal structures, cultured rudiments were stained with laminin to visualize the basal membrane and with DBA-lectin to visualize the UB (Fig. 4.A,B). The cultured kidney rudiments underwent the different morphological structures, known for *in-vivo* kidney development, thus offering a powerful system for *ex-vivo* studies.

### Staining and Localization of Proteins involved in Kidney Development

**Figure 5:**
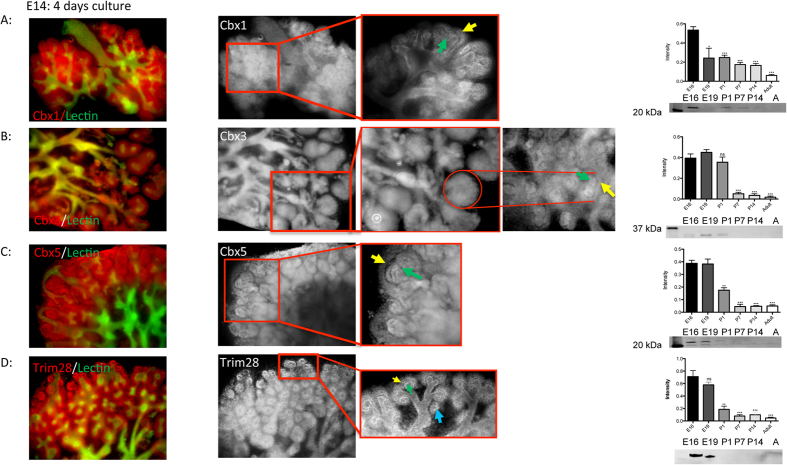
Staining of heterochromatin protein 1. (**A**) Immunofluorescence co-staining of Cbx1 (red) and DBA lectin (green) in the cultured kidneys and enlarged section of the Cbx1 staining in black and white. Cbx1 is expressed in the metanephric mesenchyme and at the tips of the ureteric bud Western blot analysis showed a decreasing expression of Cbx1 during the development. (**B**) Immunofluorescence staining of Cbx3 (red) and ureteric bud (green) in the cultured kidneys and enlarged section of the Cbx3 staining in black and white. Cbx3 is mainly expressed in the renal vesicles, and in the comma- and s-shaped bodies, but was also present in the ureteric bud. Western Blot analysis showed high expression of Cbx3 until P1. (**C**) Immunofluorescence staining of Cbx5 (red) and lectin (green) in the cultured kidneys and enlarged section of the Cbx5 staining in black and white. Cbx5 showed expression in the vesicles, comma- and s-shaped bodies, and metanephric mesenchyme, whereas less expression of the protein was observed in the uretic bud. Western blot analysis showed high expression of Cbx5 until E19. (**D**) Immunofluorescence staining of Trim28 (red) and lectin (green) in the cultured kidneys and an enlarged section of the Trim28 staining in black and white. Trim28 was expressed in the ureteric bud and metanephric mesenchyme as well. Trim28 expression decreases significantly in the course of kidney development, and was highly expressed in the cap mesenchyme surrounding each UB tip and in the interaction interface between the metanephric mesenchyme and ureteric bud tips. Western blot analysis showed high expression of Trim28 in early stages. Yellow arrows show the cap mesenchyme, green arrows show the ureteric tip and the blue arrow shows the renal vesicle. The Western blot quantification is presented as a bar chart with error bars. Each bar represents the intensity means ± s.d. of blots from 3 independent experiments. Significant differences: *p < 0.05, **p < 0.01, ***p < 0.001. P1: new born, P7: 7 days old rats, P14: 14 days old rats, (A) adult. The full-length blotting images are presented in Supplemental Fig. 8.

The proteomics data highlighted the heterochromatin proteins as down regulated in the course of kidney development. The proteins of the HP1 family, Cbx1, Cbx3, and Cbx5 as well as Trim28, were stained with fluorescence antibodies in cultured kidneys. Cbx1 was highly expressed in the MM and at the tip of the UB, whereas the rest of the UB showed low expression of Cbx1 (Fig. 5A). Cbx3 was mainly present in the renal vesicles and in the comma- and S-shaped bodies, but was also detected in the UB (Fig. 5B). Cbx5 showed similar expression in the vesicles, comma- and S-shaped bodies, and MM, whereas less expression of the protein was observed in the UB (Fig. 5C). Trim28 was expressed in the UB and MM as well. When comparing Trim28 at E14 and later stages, its expression decreased in the course of kidney development. Moreover, in E14 Trim28 was highly expressed in the cap mesenchyme and in the interaction interface between MM and UB tips (Fig. 5D). This expression pattern was also observed in advanced stages of kidney development. Interestingly, the staining of kidney rudiments cultured for more than five days revealed that the Trim28 expression was restricted to UB and the branching area and to a less amount in renal vesicle, but it was not observed in comma- or S-shaped bodies (Fig. 5D).

### Impact of Heterochromatin Protein 1 knockdown on Embryonic Kidney Development

**Figure 6:**
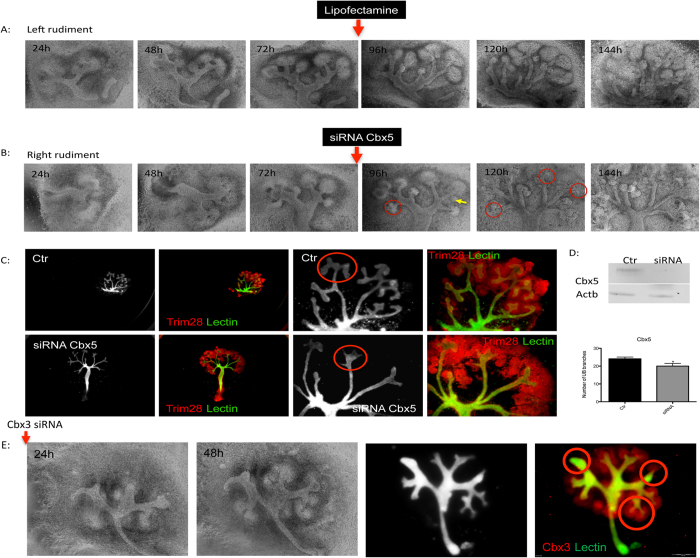
Impact of Cbx5 knockdown on kidney development. Kidney rudiments (2–4 UB tips) were cultured for 48–72 h to make sure that the kidneys attached and developed normal. The right kidney rudiment was then transfected with siRNA against Cbx5, whereas the left rudiment was given media containing only lipofectamine. The siRNA treated rudiments and controls were grown for additional 72 h. (**A**) The temporal development of the left rudiment showing a normal growth of the cultured kidney. (**B**) The temporal development of the right rudiment before and after Cbx5 siRNA application. (**C**) The kidneys were stained with immunofluorescence stain to visualize Trim28 and with DBA lectin to visualize the ureteric bud. (**D**) The knockdown was confirmed by Western blot and led to an abnormal growth of the ureteric bud and in slight but significant impairment of branching. (**E**) Impact of Cbx3 knockdown on the kidney development. Kidney rudiments (2–4 UB tips) were cultured for 48–72 h to make sure that the kidneys attached and developed normal. The right kidney rudiment was then transfected with siRNA against Cbx3, whereas the left rudiment was given media containing only lipofectamine. The siRNA treated rudiments and controls were grown for additional 48 h. Cbx3 knockdown resulted in significant impairment of kidney growth and branching arrest (red Cbx3, green DBA lectin). The branching quantification is presented as a bar chart with error bars. Each bar represents the branches number means ± s.d. from 10 cultured rudiments for siRNA and 10 for control. Significant differences: *p < 0.05, **p < 0.01, ***p < 0.001.

**Figure 7:**
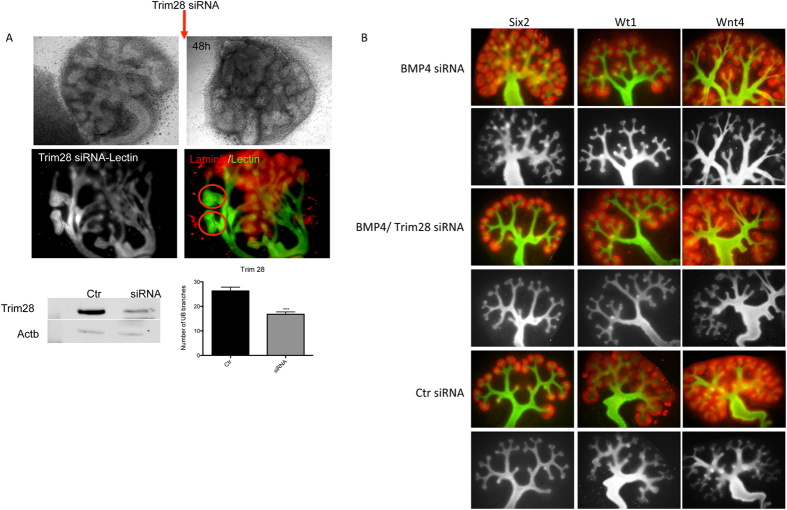
(**A**) Impact of Trim28 knockdown on kidney development. Kidney rudiments (2–4 UB tips) were cultured for 48–72 h to make sure that the kidneys attached and developed normal. The right kidney rudiment was then transfected with siRNA against Trim28, whereas the left rudiment was given media containing only lipofectamine. The siRNA treated rudiments and controls were grown for additional 48 h. The temporal development of the left rudiment showed a normal growth of the cultured kidney, whereas the Trim28 knockdown resulted in significant impairment of kidney branching and growth (red laminin, green DBA lectin). Western blot analysis showed more than 60% knockdown of Trim28, the full-length blotting images are presented in Supplemental Fig. 9. The reduced expression of Trim28 led to significant reduction of ureteric bud branching, and in some cases to growth arrest. The branching quantification is presented as a bar chart with error bars. Each bar represents the branches number means ± s.d. from 10 cultured rudiments for siRNA and 10 for control. Significant differences: *p < 0.05, **p < 0.01, ***p < 0.001. (**B**) The Bmp4 knockdown seems to rescue the effect of Trim28 knockdown. The siRNA (for Bmp4 and Trim28) treated rudiments and siRNA control treated rudiments were grown for additional 48 h. Bmp4 knockdown rudiments showed an enhanced branching along the ureter as evidenced by lectin (black and white) and Six2 Wt1 and Wnt4. The combined Trim28/Bmp4 knockdown did not show any impairment of the kidney branching revealing potential rescue of Trim28 knockdown effect by Bmp4 knockdown. (red Six2, Wt1, Wnt4, green DBA lectin).

To investigate the role of Cbx1, Cbx3, Cbx5 and Trim28 in kidney development, *ex-vivo* knockdowns in cultured kidney of the given protein using psiRNA and siRNA were performed. Before performing the knockdown in kidney rudiments the psiRNA constructs were tested on human kidney cells (TK173) and murine embryonic stem cells. The knockdown achieved was almost 100% of Trim28 in TK173 and >50% in murine embryonic stem cells (Supplemental Fig. 5). On the kidney rudiments, the knockdowns with these constructs were performed directly on the embryonic kidneys as described in methods (Supplemental Fig. 6A). Due to the GFP insert in the plasmid used, it was possible to follow the efficiency of the transfection in the kidney, as seen in the case of transfection with Cbx5 and Trim28 (Supplemental Fig. 6B). The efficiency of the knockdown was monitored using fluorescence staining. The down-regulation of the proteins, especially Trim28 showed obvious effects on the kidney development, mainly on the UB branching and nephron formation. The use of psiRNA demonstrated that the down-regulation of heterochromatin protein 1 had an impact on the kidney development. To further increase the efficiency of the down-regulation, siRNA was applied directly to the kidney rudiments. Kidney rudiments (2–4 UB tips, n = 20) were cultured and the UB tips grown to 8 ± 2.6 UB. Thereafter, every left kidney rudiment was transfected with siRNA against Cbx5 (n = 10), whereas the right kidney rudiment was kept as control (n = 10). The transfected kidneys and controls were then grown for additional 72 h. Western blot analysis revealed a significant knockdown of the proteins (Fig. 6). Interestingly knockdown of Cbx5 resulted in an abnormal growth of the UB (Fig. 6A–C) and in slight but significant decrease in UB branching (Fig. 6D). The knockdown of Trim28 led to an expression reduction of more than 60% of the protein (Fig. 6D) as evidenced by Western blot analysis. In case of Cbx3, knockdown of the protein resulted in reduction in the number of ureteric branches and even in kidney growth alteration (Fig. 6E). The decrease in Trim28 expression resulted in similar effect as in case of Cbx3, significant reduction of UB branching in some cases even to UB branching arrest (Fig. 7A). Knockdown of Bmp4, a target transcription factor of Trim28, seemed to enhance branching along the UB as evidence by staining of marker of kidney development (Fig. 7B). A combined Trim28/Bmp4 knockdown seemed to rescue the effect of Trim28 as well as Bmp4 knockdown (Fig. 7B) revealing that the effect of Trim28 on kidney development is mediated by Bmp4. Knockdown of Cbx1 did not show any significant effect on the kidney development (data not shown).

### ChIP Analysis of H3K9me3 in the Developing Kidney

**Figure 8:**
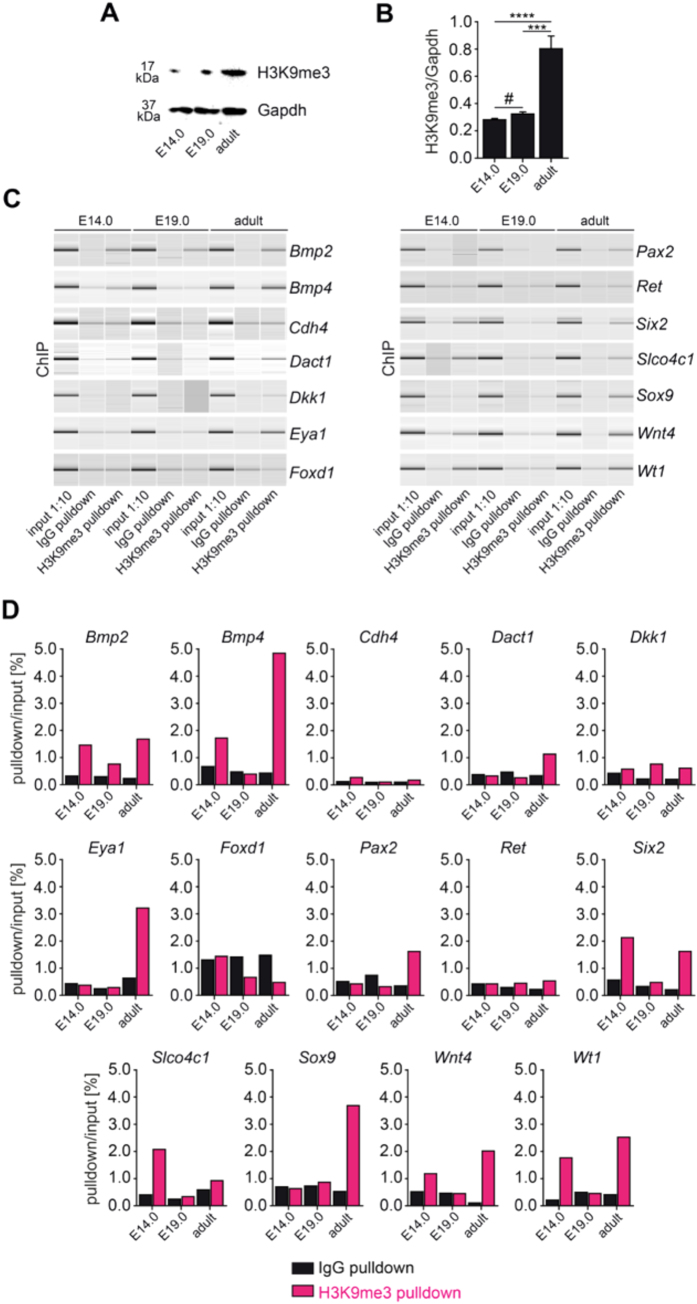
Kidney development is associated with dynamic changes in H3K9me3 distribution. (**A,B**) As determined by SDS-Page and subsequent immunoblotting using total kidney lysates from different stages of kidney development, total levels of H3K9me3 normalized to Gapdh remained unchanged between E14 and E19, whereas increased levels were observed in adult kidneys (data are presented as means ± sd, measurements were done in technical triplicates, ***p < *0.001*, ****p < *0.0001*, ^#^*no significance*, values of *p* were calculated using one-way ANOVA with Bonferroni post-hoc analysis respective to E14). (**C,D**) PCR products from input samples diluted 1 to 10 (input 1:10), IgG pulldowns as negative controls and DNA pulldowns using H3K9me3 antibody (H3K9me3 pulldown) at different stages of kidney development (E14, E19 or adult) were analyzed on a Bioanalyzer, gel images and DNA measurements are shown respective to input samples. Alterations in H3K9me3 distribution between E14 and E19 could be identified for *Bmp2, Bmp4, Six2, Slco4c1, Wnt4* and *Wt1*.

**Figure 9:**
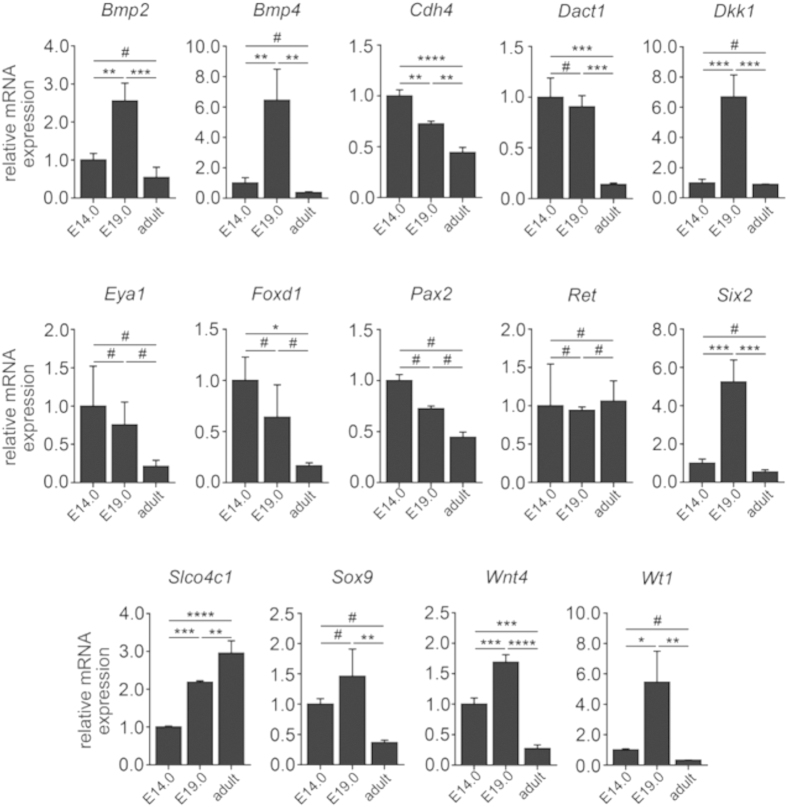
*Bmp2, Bmp4, Six2, Slco4c1, Wnt4* and *Wt1* show dynamic changes during kidney development. As analyzed by qRT-PCR from RNA extracted from total kidney lysates, *Bmp4, Six2, Slco4c1, Wnt4* and *Wt1* mRNA expression levels correlated inversely with H3K9me3 distribution among gene promoters of *Bmp4, Six2, Slco4c1, Wnt4* and *Wt1* (data are presented as means ± s.d., measurements were done in technical triplicates, *p < *0.05*, **p < *0.01*, ***p < *0.001*, ****p < *0.0001*, ^#^*no significance*, values of *p* were calculated using one-way ANOVA with Bonferroni post-hoc analysis respective to E14.0).

Because Trim28 and HP1 are involved in recognition of repressive H3K9me3 histone mark and mediates gene silencing and heterochromatin formation^36^,^37^, we analyzed H3K9me3 levels in total kidney lysates at embryonic day 14 (E14) and 19 (E19), adult kidneys were included for comparison. As determined by immunoblotting, H3K9me3 was detected during kidney development with an increase in adult kidneys (Fig. 8A,B). To gain insights into potential silenced gene by Trim28 and Cbx5, gene promoters of factors involved in kidney development (*Bmp2, Bmp4, Cdh4, Dact1, Dkk1, Eya1, Foxd1, Pax2, Ret, Six2, Slco4c1, Sox9, Wnt4, and Wt1*) were analyzed by chromatin immunoprecipitation (ChIP). Although total H3K9me3 levels remained unchanged between E14 and E19 (Fig. 8A,B), we found dynamic changes in the distribution of the gene promoters bound to H3K9me3 between E14 and E19 especially for the following genes: *Bmp2, Bmp4, Six2, Slco4c1, Wnt4* and *Wt1* (Fig. 8C,D). This is in accordance with the public H3K9me3 ChIP-seq datasets from human fetal kidneys (GSM621441), which also identified enrichment of H3K9me3 at these gene promoters. Consistent with this finding, repressive H3K9me3 distribution at these gene promoters at E14 and E19 inversely correlate with mRNA expression levels analyzed by qRT-PCR (Fig. 9), potentially reflective of an involvement of Trim28/HP1/H3K9me3 in transcriptional regulation of the genes controlling kidney development.

## Discussion

During kidney development the interactions between epithelial cells and their neighboring mesenchyme cells are of particular importance. This reciprocal interaction is central in kidney morphogenesis and in formation of the functional kidney unit nephron^4^. Although the mechanisms behind nephrogenesis have been investigated extensively the last decades^4^, the signals that trigger and control UB branching morphogenesis remain unclear. Comparative proteomics analyses performed in this study suggest developmental stage specific pathway activation. The functional annotation of the identified proteins proposes a group of the identified proteins to be involved in embryonic organ development (Aldh1a1, Rbp4, Aldh1a2, Aldh1a3, Krt8, and Sod1) and in kidney development (Aldh1a1, Aldh1a2, Aldh1a3, and Aldh9a1). Particularly the proteins found to be involved in kidney development are important candidates in the retinoic acid (RA) pathway, which is known to play a central role in renal development. RA is known to control the embryonic kidney patterning by modulating UB branching^38^; it controls the expression of Ret, a tyrosine kinase receptor, and a key molecule in the RA pathway. Ret modulates the ureteric bud branching morphogenesis^39^. Aldh1a1 and Aldh1a2 belong to the aldehyde dehydrogenase enzyme family, which include 17 genes. Aldh1a1 and Aldh1a2 catalyze locally the oxidation of retinal to RA, which in turn can affect biological processes^40^. Aldha1a1 has been reported to be a stem cell marker^41^,^42^. The Aldh1a2 knockout mouse suffer from severe RA deficiency and show developmental abnormalities^43^. Once in the cell, retinol is converted into retinoic acid. Crabp1 and Crabp2 are important for the transport of RA from the cytosol to the nucleus where it serves as a ligand for nuclear retinoic acid receptors (RARs) that directly regulate gene transcription^44^, especially of genes that modulate the overall development of the embryo. The regulation of the RA-pathway proteins during embryonic development emphasizes the importance of this pathway for renal development.

We showed that in an early stage of kidney embryonic development Trim28 is highly expressed in the cap mesenchyme and at the tips of the UB, while it is of minor abundance in the UB. Our data revealed that during the MET Trim28 is expressed in the pretublar aggregate and renal vesicle, whereas no Trim28 expression was detected in comma- and S-shaped bodies and in the elongated nephrons. The high expression of Trim28 in the branching area of the embryonic kidney and the fact that knockdown of Trim28 resulted in branching arrest suggest that Trim28 plays an important role in kidney branching and morphogenesis.

Trim28 is a tripartite motif-containing 28 protein, which is involved in transcriptional control of many zinc finger proteins transcription factor (Zfp) with the krüppel-associated box repression domain (Krab). Trim28 binds to DNA via Krab Zfp and acts as a molecular scaffold that controls gene-specific silencing. In a previous work^30^ we showed that Trim28 is highly expressed in embryonic and multipotent adult germline stem cells, and that the stem cell differentiation was accompanied by decrease in Trim28 expression. Trim28 is required for proper oocyte to embryo transition^45^ and for the maintenance of imprinting marks immediately after fertilization^46^,^47^,^48^. Trim28 is essential for maintaining pluripotency in stem cells, which will die or undergo differentiation if removed^49^,^50^, on the other hand Trim28 was found to be necessary for mouse embryonic stem cells^51^,^52^ and for macrophage differentiation^53^. Trim28 knockdown lead to increased cell proliferation, whereas overexpression of Trim28 lead to decreased cell proliferation^54^. It has been established that Trim28 through Setdb1 is responsible for maintaining endogenous retroviruses (ERVs) in a silent state in ES cells and early embryos^49^,^55^ and an important role of this process is to protect the transcriptional dynamics of early embryos from perturbation by cis-acting activators contained in these mobile elements^56^. O’Geen *et al.*^57^ performed a genome-wide ChIP-chip (chromatin immunoprecipitation followed by DNA microarray) analysis of Trim28 binding sites throughout the human genome using a 38-array tiling set. The authors identified ~7000 Trim28 binding sites. To understand to role of the repressive function of Trim28 in the kidney development, we performed bioinformatics wide analyses of O’Geen *et al.* data using DAVID bioinformatics. The identified ~7000 binding sites resulted in 1687 DAVID IPs, among these genes 22 are involved in kidney development, (e.g. Wt1, Bmp4, Bmp7, Gdnf, and Ret) and their expression seems to be under the control of Trim28. The UB branching is controlled by a number of signaling molecules, of which several growth factors, such as Bmps, Gdnf and Fgfs have been described to play important roles in this process^16^,^17^,^21^,^22^,^23^. Reciprocal signaling between UB and the MM via Gdnf and its receptor Ret is the major factor for the outgrowth of the UB and further budding, as well as the interaction between Bmp4 and Gremlin. Bmp4 is expressed in stromal cells, which cover the nephric duct and was described to be responsible for inhibition of UB outgrowth^58^. Bmp4 knockout mice die in early development stages, whereas the heterozygous mice develop ectopic budding^59^,^60^,^61^. Bmp4 is also known to block the Gdnf induced UB branching through Smad1 activation^62^. Bmp4 itself can be neutralized through binding of its antagonist Gremlin^21^,^63^,^64^. Bmp4 is one of the genes, which were identified as potentially repressed by Trim28. We suggest that the high expression of Trim28 in the UB tips is a part of the targeted UB branching control mechanisms. The repression of Bmp4 by Trim28 could be a complementary process to the antagonizing effect of Grem1 and indeed blocking the negative effect of Bmp4 on UB branching. This is supported by the fact that the down-regulation of Trim28 resulted in branching arrest in cultured kidney. Another aspect, which highlights the role of Trim28 in kidney development, is the absent expression of the protein in elongating nephron and in almost whole kidney in late stages. The ChIP analyses, performed by O’Geen *et al.*, revealed a potential interaction between Trim28 and key transcription factors of MET and nephron elongation. Wnt11, Wt1, Bmp7, Jag1 and Gdnf belong to the potential transcription factors that can be repressed by Trim28. Indeed down-regulation of Trim28 in renal vesicle could favor the expression of key transcription factors for MET and nephron elongation. In late stage of the development the kidney reach the right size and branching should stop. This could be promoted by down-regulation of Trim28.

HP1 builds a family of proteins that bind histone H3 methylated on lysine 9 (H3K9). HP1 plays an important role in heterochromatin formation and is typically involved in the epigenetic regulation of gene silencing^65^. However, recent reports have demonstrated that HP1 can activate gene expression in certain contexts including differentiation^66^. The mechanism behind gene expression activation of HP1 is still poorly understood. The proteomic screening of embryonic kidney development revealed a significant expression alteration of three HP1 isoforms β, γ and α (Cbx1, Cbx3 and Cbx5 respectively). To explore the role of the HP1 proteins Cbx1, Cbx3 and Cbx5, in kidney development, their expression was individually disrupted in cultured kidney rudiments and the kidney branching and differentiation were monitored. Among these three isoforms, Cbx5 and Cbx3 were required for normal development of the kidney in early stages, whereas Cbx1 knockdown did not affect rudiment kidney branching and morphogenesis. The knockdown of Cbx3 resulted in branching alteration, whereas the expression alteration of Cbx5 led to uncontrolled growth of the UB and slightly alteration in UB branching. As interaction partner of Trim28, the HP1 proteins can act in concert with Trim28. In case of Cbx5 the uncontrolled growth of UB second to knockdown suggests that Cbx5 is involved in silencing of key proteins involved in UB growth. More investigations are required to explore the genes, which are under HP1 control and which play a central role in kidney development.

To unravel the protein expression changes that accompany the kidney development transcriptomic analysis offer a useful method, which is widely used for comparison of gene expression of different biological samples. Nonetheless, mRNA is subjected to the impact of many factors, which can significantly influence the amount of protein synthesized. Additionally, post-transcriptional and post-translational modifications, which play important roles in embryonic development, increase the diversity of proteins that can be synthesized from a fixed number of genes. Additionally, changes in gene expression, which are accompanied by alteration in mRNA level, do not always result in protein expression or activity modification. Proteome investigation often helps to overcome the limitations of the transcriptomic analysis but depending on the methods used the proteome analysis has also some limitations. For example the 2-DE used in our study has several limitations as a separation method: hydrophobic proteins hardly enter the gel and are often lost during 2-DE, limiting its use for the analysis of e.g. integral membrane proteins. Very high or very low molecular weight proteins, highly acidic or highly basic proteins may also be lost. Due to the often limited staining sensitivity, 2-DE also requires relatively large amounts of protein.

## Conclusions

In this study, we presented a comprehensive proteomics mapping of kidney embryonic development. Interestingly, heterochromatin proteins known to be involved in the epigenetic regulation of gene silencing were indispensible for keeping the balance between branching activators and inhibitors in early stage of development.

## Methods

### Animals

Wistar Han rats were kept under 12:12 h cycle of light with *ad libitum* access to food and drink. The rats were sacrificed at embryonic day 14 (E14), 16 (E16), and 19 (E19), and at postnatal day 1 (P1) and the kidneys were dissected from the embryos as well as from neonatal pups. To assure for biological replication 5–8 pregnant rats were used per embryonic stage. The kidney protein extracts were prepared from 95 kidneys at stage E14, 60 at E16, 40 at E19, and 20 kidneys were used for the P1 protein extract. All experimental procedures were performed according to the German animal care and ethics legislation (NIH standards) and were approved by the local Ethics Committee of the University Medical Centre Göttingen, Germany.

### Protein Extraction

For protein extraction for 2-D gel electrophoresis (2-DE), the kidneys from embryos at the same embryonic stage and female (14–17 embryos) were pooled, disrupted with lysis buffer (9.5 M urea, 2% CHAPS (w/v), 2% ampholytes (w/v), 1% DTT) and vortexed. After adding the lysis buffer, the samples were incubated for 30 min at 4 °C. For removing the cell debris, centrifugation was carried out at 13,000 × g and 4 °C for 30 min. The supernatant was recentrifuged at 13,000 × g and 4 °C for an additional 30 min to get maximal purity. The pellet was discarded, and the resulting samples were used immediately or stored at −80 °C until use.

### Protein Precipitation

To reduce the salt contamination and to enrich the proteins, methanol-chloroform-precipitation according to Wessel and Flugge^67^ was performed. The pellet was dried and dissolved in lysis buffer. Total protein concentration was determined using the Bio-Rad protein assay (Bio-Rad, Hercules, CA, USA) according to Bradford^68^. BSA (Sigma, Steinheim, Germany) was used as a standard. Total protein concentration was determined using the Bio-Rad protein assay (Bio-Rad, Hercules, CA, USA) according to Bradford^68^. BSA (Sigma, Steinheim, Germany) was used as a standard.

### 2D Gel Electrophoresis (2-DE) and Gel Staining

The 2D protein separation was carried out as described earlier^30^. The 2-DE gels were stained with Flamingo fluorescent gel stain (Bio-Rad, Hercules, CA, USA) following the manufacturer instructions. After staining, gels were scanned at 50 μm resolution on a Fuji FLA-5100 scanner. The digitalized images were analyzed using Delta 2D 3.4 (Decodon, Brunswick, Germany). For protein visualization, the 2-DE gels were additionally stained overnight with colloidal Coomassie blue, Roti-Blue (Roth, Karlsruhe, Germany).

### 2D-DIGE

For 2D-DIGE, protein extraction and methanol-chloroform-precipitation were performed as described above. The resulting pellet was dissolved in labeling buffer (30 mM Tris-HCl pH 8.5, 9.5 M urea, 2 % CHAPS), centrifuged, and the protein concentration of the supernatant was determined as described above. The preparation of the CyDyes and the labeling reaction were performed according to the manufacturer’s protocol (GE Healthcare). To control for dye-specific protein labeling, every pair of protein samples from two independent kidney extract preparations were processed in duplicate while swapping the dyes. Thereby four replicate gels were obtained, allowing monitoring regulation factors down to twofold changes. The CyDye-labeled gels were scanned at 50 μm resolution on a Fuji FLA5100 scanner (Fuji Photo, Kanagawa, Japan) with laser excitation light at 473 nm and long pass emission filter 510LP (Cy2), 532 nm and long pass emission filter 575LP (Cy3), and 635 nm and long pass emission filter 665LP (Cy5). Fluorescent images were acquired in 16-bit TIFF files format. Spot matching across gels and normalization based on the internal standard was performed with Delta2D software (Decodon, Greifswald, Germany). To analyze the significance of protein regulation, a Student’s *t*-test was performed, and statistical significance was assumed for p-values less than 0.01. For protein visualization, the 2-DE gels were post-stained with colloidal Coomassie blue (Roti-Blue) overnight. Differentially regulated proteins were excised and processed for identification by mass spectrometry.

### Protein Identification

Manually excised gel plugs were subjected to an automated platform for the identification of gel-separated proteins^69^ as described. An Ultraflex MALDI-TOF-TOF mass spectrometer (Bruker Daltonik) was used to acquire both PMF and fragment ion spectra, resulting in confident protein identifications based on peptide mass and sequence information. Database searches in the Swiss-Prot and NCBI primary sequence database restricted to the taxonomy *rattus norvegicus* were performed using the MASCOT Software 2.2 (Matrix Science). Carboxamidomethylation of Cys residues was specified as fixed and oxidation of Met as variable modifications. One trypsin missed cleavage was allowed. Mass tolerances were set to 100 ppm for PMF searches and to 100 ppm (precursor ions) and 0.7 Da (fragment ions) for MS/MS ion searches. The minimal requirement for accepting a protein as identified was at least one peptide sequence match above identity threshold in addition to at least 20% sequence coverage in the PMF.

### Bioinformatics

The classification of the identified proteins according to their main known/postulated functions was carried out using DAVID bioinformatics^26^. The official gene symbol was used to investigate and categorize the gene ontology (GO)-annotations (biological processes and molecular functions). Network analysis of known and predicted protein-protein interactions of the identified proteins were performed using STRING (search tool for the Retrieval of Interacting Genes/Proteins) 9.1^70^. The interaction data from direct and indirect associations derived from genomic context, high-throughput experiments, coexpression and previous knowledge for a large number of organisms are integrated in this database.

### Western Blot Analysis

Proteins (40 μg) were separated by SDS-PAGE and transferred to Hybond ECL nitrocellulose membrane (GE Healthcare). Immunodetection was performed according to Towbin *et al.*^71^. Mouse monoclonal anti-Trim28/Kap1 [20C1] antibody ChIP Grade (Abcam), rabbit monoclonal anti- Cbx5/HP1 alpha antibody (Abcam)/ goat polyclonal to Cbx5/HP1 alpha-ChIP Grade (Abcam), rabbit polyclonal to Cbx1/HP1 beta (Abcam), rabbit polyclonal to Cbx3/HP1 gamma (Abcam), mouse monoclonal anti-ß-actin (Sigma) (1:5000), rabbit polyclonal anti-H3K9me3 ChIP grade (Abcam) and Gapdh (HyTest, Turku, Finland) were used as primary antibodies. Molecular Probes Alexa Fluor 647 goat anti-mouse IgG antibody or Alexa Fluor 647 goat anti-rabbit IgG (1:2000) were used as secondary antibodies. Before imaging, the blots were dried in the dark. The blot membranes were scanned at 50 μm resolution on a Fuji FLA-5100 scanner (Fuji Photo) with single laser-emitting excitation light at 635 nm.

### *Ex-vivo* Organ Culture of Embryonic Kidney

The *ex-vivo* organ culture was carried out according to Davies^12^. Embryonic kidneys at day E14 were excised and plated on a transparent PET membrane with 0.4 μm pore size (24 well, BD Falcon). The membrane was placed in one well of a 24 well plate, containing 400 μl kidney culture medium (KCM, DMEM, 10% inactivated FCS). This enabled the kidney to get in touch with the medium without drowning it. Under these conditions it was possible to keep the kidney cultured for at least 144 h at 37 °C and 5% CO_2_.

### RNAi Knockdown

The excised kidneys were transfected with psiRNA-h7SKGFPzeo plasmid containing short-hairpin (sh) RNA carrying sequences representing targets on the mRNA of the proteins of interest (Cbx1: GGATTATTGG AGCTACTGAC T; Cbx3: GAACATGAAG TGTCCTCAGA T; Cbx5: GGCAAGTGGA ATATCTGTTG A). The kidneys were incubated in a transfection suspension containing lipofectamine (Invitrogen) and the psiRNA for 3 h at 37 °C under shaking, before it was placed on a membrane in the transfection suspension overnight to grow as described above. The next day the medium was changed to KCM, and the kidney could be kept in culture for at least 4–6 days before fixation in methanol and following immunofluorescence staining.

### siRNA Transfection

For siRNA transfection the excised kidney rudiments were placed on PET membrane and cultured for 48–72 h to make sure that the kidneys attached and developed normal. Thereafter the transfection suspension containing lipofectamine (Invitrogen) and siRNA targeting the mRNA of interest was added to the rudiment (Supplemental Table 2). The kidney was incubated in the transfection suspension overnight to grow as described above. The next day the medium was changed to KCM, and the kidney was kept in culture up to six days before fixation in methanol and following immunofluorescence staining.

### Histochemical Staining

For histological staining of the embryonic kidneys, the excised kidneys were fixed overnight in 4% buffered formalin solution, and subsequently embedded in paraffin blocks. The paraffin embedded sections were deparaffinized and rehydrated, and stained with hematoxylin solution mod. Acc. To Gill III and eosin Y solution (Merck, Darmstadt, Germany) according to manufacturer’s protocol.

### Immunofluorescence Staining

After culturing the kidney for the desired amount of time, they were fixed in cold methanol at −20 °C overnight, washed 3 times in PBS before the primary antibody (mouse monoclonal anti-Trim28/Kap1 [20C1] antibody ChIP Grade (Abcam), rabbit monoclonal anti-Cbx5/HP1 alpha antibody (Abcam), rabbit polyclonal to Cbx1/HP1 beta (Abcam), rabbit polyclonal to Cbx3/HP1 gamma (Abcam), Rabbit monoclonal anti-laminin antibody (Sigma)), in PBS was added and incubated at 4 °C overnight. The kidneys were washed for 4 h in PBS and the secondary antibody (Molecular Probes Alexa Fluor 555 goat anti-mouse IgG antibody, Alexa Fluor 555 goat anti-rabbit IgG or Alexa Fluor 555 donkey anti-goat IgG (1:500)) was added overnight at 4 °C. The UB was stained using lectin *Dolichos biflorus* agglutinin (DBA) for at least 3 h. After incubation, the kidneys were washed for 3 h in PBS and embedded for microscopic analysis.

### RNA Isolation

Tissues were dissolved in TRIzol (Invitrogen, Carlsbad, USA) and shredded using TissueLyser LT (Qiagen, Hilden, Germany). RNA was isolated using PureLink RNA Mini Kit (Ambion, Carlsbad, USA) according to the manufacturer’s protocol.

### Quantitative Real-Time PCR Quantification (qRT-PCR)

For SYBR-based real-time PCR, cDNA synthesis from 500 ng total RNA was performed using DNase I treatment and SuperScript II Reverse Transcriptase (Invitrogen, Carlsbad, USA) according to the manufacturer’s protocol. 1 μl of reverse-transcribed cDNA was added to the reaction mixture containing the primer pair (200 nmol/l each) (Supplemental Table 3) and diluted 2× Fast SYBR Green Master Mix (Applied Biosystems, Carlsbad, USA) in a final volume of 20 μl for each PCR reaction. The real-time PCR reactions were performed in a 96-well reaction plate using the StepOne Real-Time System (Applied Biosystems, Carlsbad, USA) and were done in triplicates. An initiation step at 95 °C for 20 seconds was followed by 40 cycles at 95 °C for 3 seconds and 60 °C for 30 seconds, with one cycle of dissociation at 95 °C for 15 seconds, 60 °C for 60 seconds, and 95 °C for 15 seconds. The intercalation of SYBR Green dye and its fluorescent signal is directly proportional to the amount of amplified DNA and was transformed into the cycle threshold (Ct). For normalization, the Ct values of the housekeeping gene were subtracted from the Ct values of the gene of interest to generate the dCt values. The relative expression levels were calculated using the equation 2^−ddCt^.

### Chromatin Immunoprecipitation (ChIP)

ChIP assay was performed using OneDay ChIP kit and Shearing ChIP kit (Diagenode, Denville, USA) according to the manufacturer’s protocol. After cross-linking, cells were fixed in formaldehyde and DNA was sheared to small fragment sizes. After incubation with specific antibodies against H3K9me3 (Abcam) and IgG as a negative control (Diagenode, Denville, USA), reversed cross-linking and proteinase K treatment were carried out. 5 μl of eluted DNA was added to the reaction mixture containing the primer pair (300 nmol/l each) (Supplemental Table 3), a ROX passive reference dye (Bio-Rad, Hercules, USA) and diluted 2× SYBR green Supermix (Bio-Rad, Hercules, USA) in a final volume of 25 μl for each PCR reaction. The real-time PCR reactions were performed in a 96-well reaction plate using the Mx3000P QPCR System (Stratagene, Santa Clara, USA). PCR reaction was stopped when the fluorescent signal increased over the threshold and electrophoresis of PCR products were done on a Bioanalyzer 2100 (Agilent Technologies, Santa Clara, USA) according to the manufacturer’s protocol. Electrophoresis results are shown as virtual gel images as described in our previous publications^72^,^73^,^74^.

### Statistical Analysis

For 2-DE the digitalized images were analyzed; spot matching across gels and normalization were performed using Delta2D 3.4 (Decodon, Brunswick, Germany). Delta2D computes a ‘spot quality’ value for every detected spot. This value shows how closely a spot represents the ‘ideal’ 3D Gaussian bell shape. Based on average spot volume ratio, spots whose relative expression is changed at least 2-fold (increase or decrease) between the compared samples were considered to be significant. To analyze the significance of protein regulation, Student’s t-test was performed, and statistical significance was assumed for P values less than 0.01.

All blots were quantified using the ImageJ software. The data were compiled with the software package GraphPad Prism, version 4. The software was used for graphical presentation and analysis by Student’s t-distribution. Results are presented as the mean ± s.d. of at least three independent experiments. Differences were considered statistically significant when p < 0.05. Ureteric bud tips and nephrons were counted manually and the data were compiled with the software package GraphPad Prism, version 4.

For ChIP and qRT-PCR one-way ANOVA with Bonferroni post-hoc analysis was used for multiple comparisons of samples to determine statistical significance. Statistical significance was defined as values of *p* < *0.05*, indicated as *p < *0.05*, **p < *0.01*, ***p < *0.001* or ****p < *0.0001*. Prism 5 software (GraphPad, La Jolla, USA) was used for statistical analysis.

## Additional Information

**How to cite this article**: Dihazi, G. H. *et al.* Proteomic analysis of embryonic kidney development: Heterochromatin proteins as epigenetic regulators of nephrogenesis. *Sci. Rep.* **5**, 13951; doi: 10.1038/srep13951 (2015).

## Supplementary Material

Supplementary Information
