# Supplementary material for: Proteomic analysis of embryonic kidney development: Heterochromatin proteins as epigenetic regulators of nephrogenesis
Source: Sci Rep. 2015 Sep 11;5:13951. doi: 10.1038/srep13951 (PMC4566080; doi:10.1038/srep13951)
Supplement: Supplementary Information [file srep13951-s1.pdf]

Proteomic analysis of embryonic kidney development: Heterochromatin proteins as epigenetic regulators of nephrogenesis

Gry H. Dihazi[1], Olaf Jahn[2,3], Björn Tampe[1], Michael Zeisberg[1], Claudia Müller[1,4], Gerhard A Müller[1], Hassan Dihazi[1]

[1] Department of Nephrology and Rheumatology, Georg-August University Göttingen, Robert-Koch-Strasse 40, D-37075 Göttingen, Germany, [2] Proteomics Group, Max-Planck-Institute of Experimental Medicine, Hermann-Rein-Strasse 3, D-37075 Göttingen, Germany, [3] Deutsche Forschungsgemeinschaft Research Center for Molecular Physiology of the Brain, Humboldtallee 23, D-37073 Göttingen, Germany, [4] Section for Transplantation- Immunology and Immunohematology, ZMF, Eberhard-Karls-University Tübingen, Germany

Corresponding Author

[*] Corresponding Author: Hassan Dihazi, Prof. Dr. rer. nat., Department of Nephrology and Rheumatology, University Medical Center Göttingen, Georg-August University, Robert-Koch-Strasse 40, D-37075 Göttingen, Germany, Telephone: +049-551-3991221, Fax: +049-551-3991039, E-mail: dihazi@med.uni-goettingen.de

Supplementary Figure S1

A:

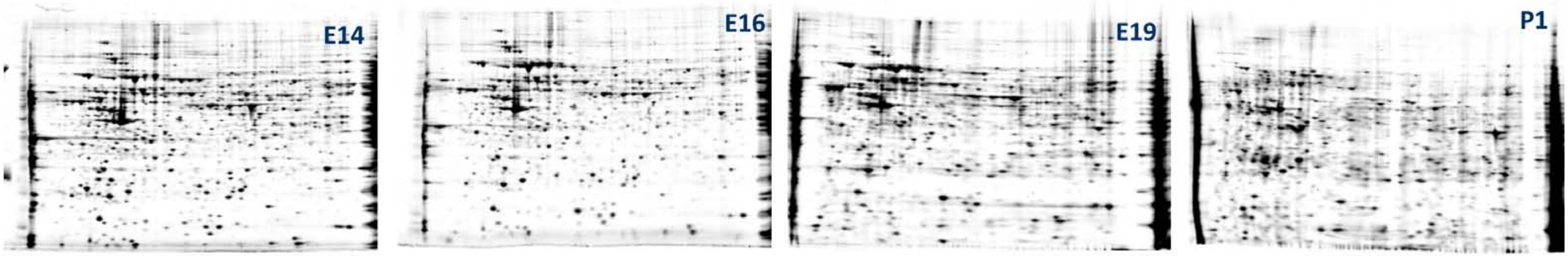

B

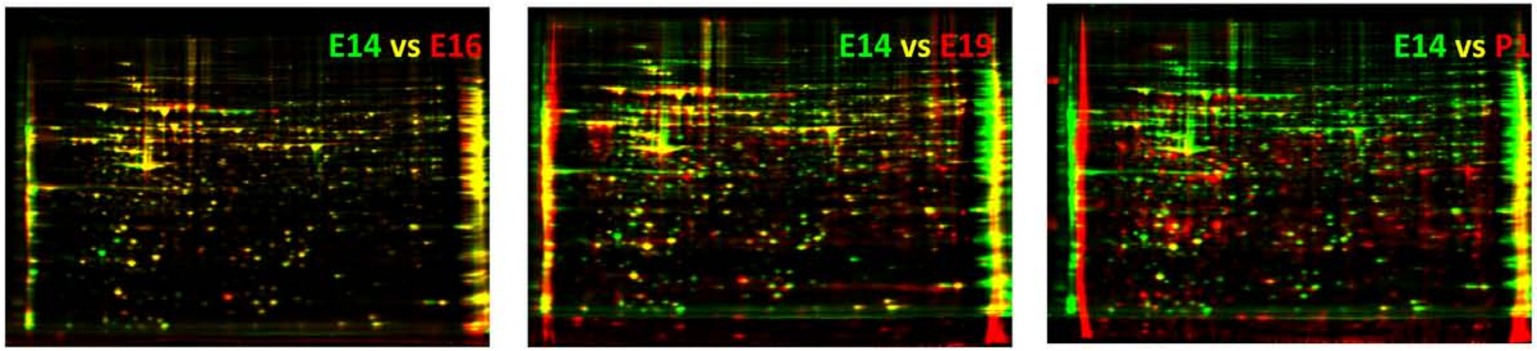

C

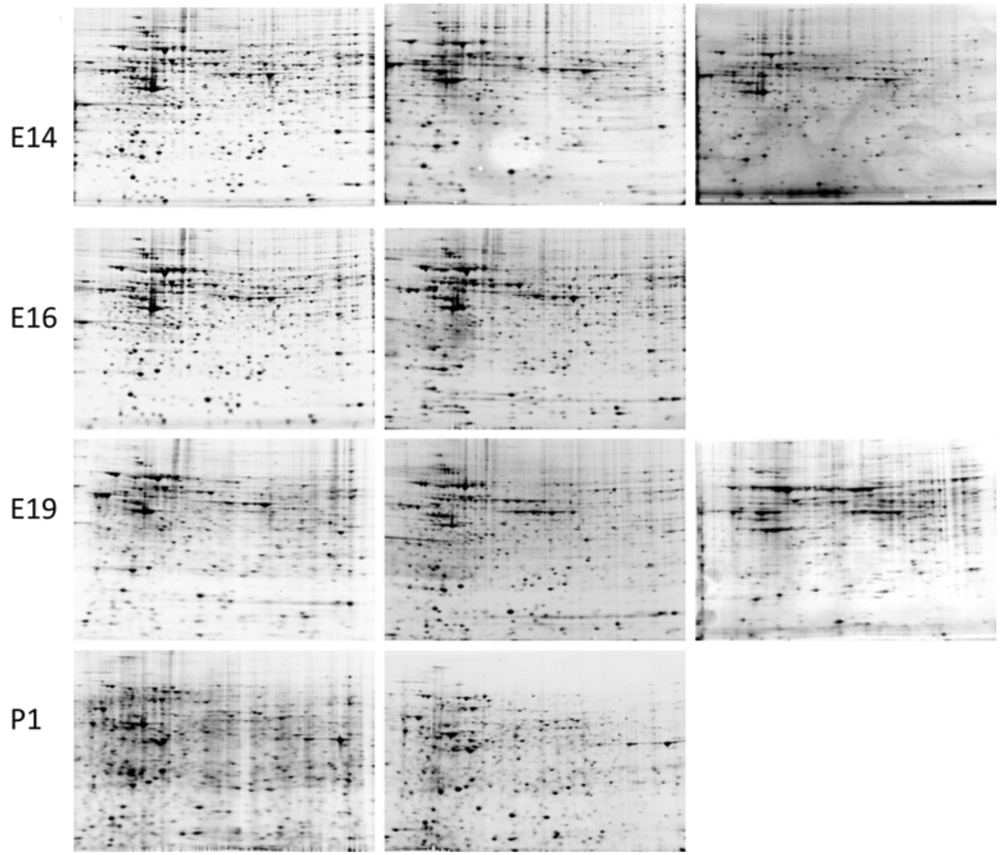

Supplemental Figure 1: A: 2-DE reference maps of proteins extracted from rat kidney at E14, E16, E19 and. 150 μg proteins were loaded on an 11-cm IPG strip with a linear pH gradient pI 5-8 for IEF, 12% SDS-polyacrylamide gels were used for the SDS-PAGE. Proteins were stained with Flamingo fluorescent gel stain. B: Overlays of the 2DE-gels. The gels were compared with Delta 2D (Decodon). The protein maps of the kidneys at the early stages E14 and E16 revealed high similarity, whereas E19 and P1 overlay display significantly different protein pattern. C: 2DE Replicates

Supplementary Figure S2

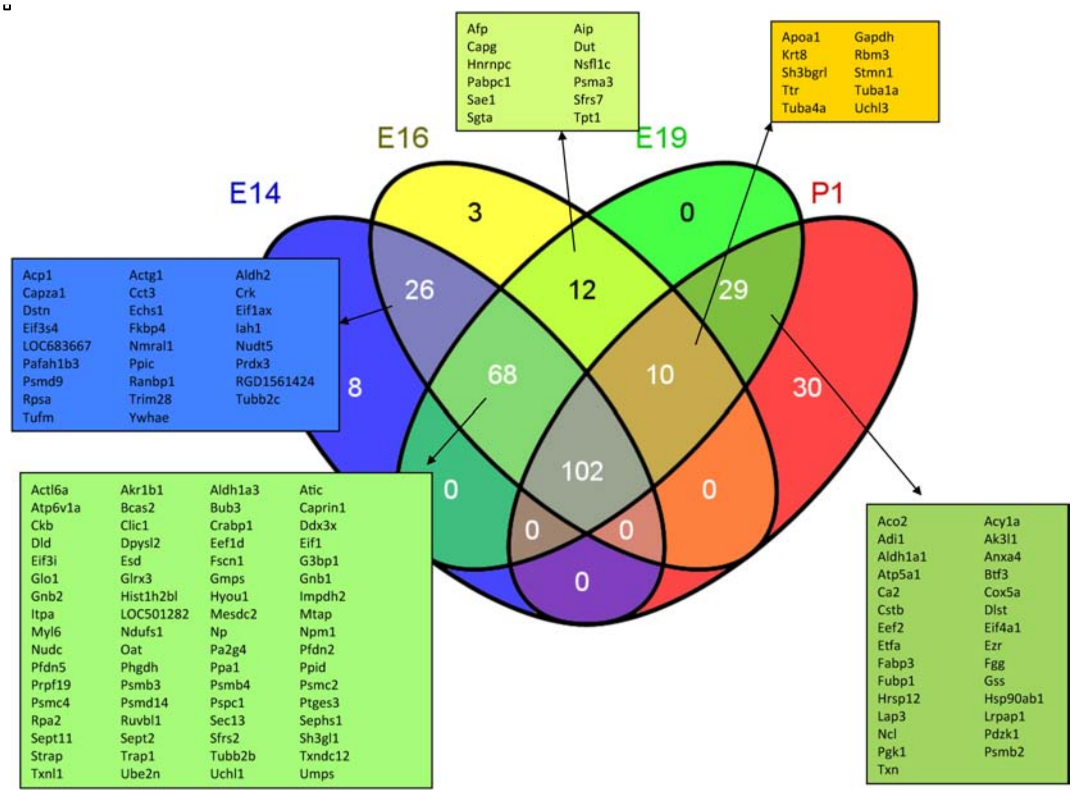

Supplemental Figure 2: Venn diagram of the identified proteins. 104 of the identified proteins were found in all developmental stages, focusing on the proteins found in two or three stages. E14 and E16 had 86% of the identified proteins in common; E19 and P1 had only 55 % common proteins, whereas E14 and P1 shared only 36 % of the identified proteins.

Supplementary Figure S3

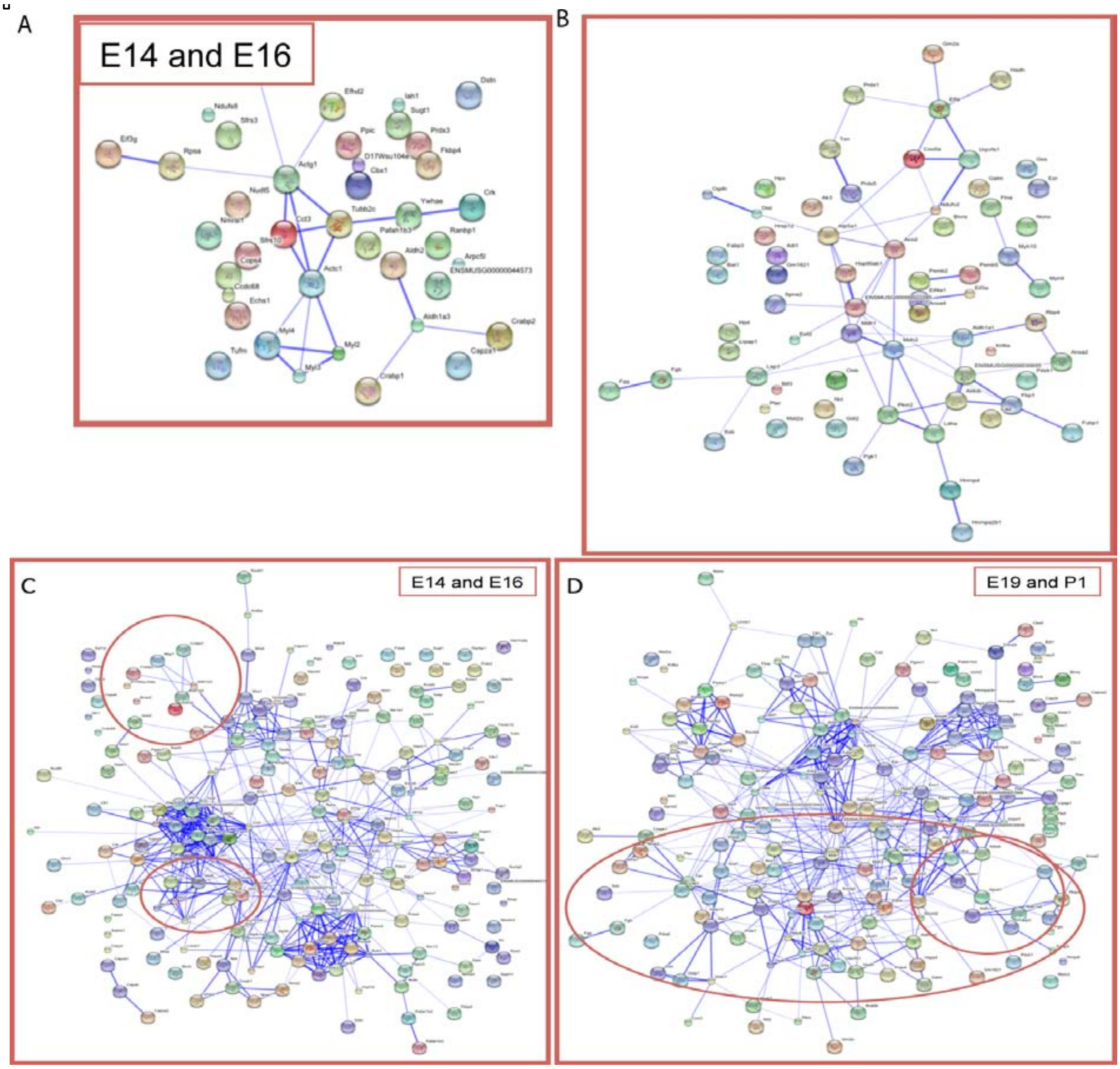

Supplemental Figure 3: A: Protein interaction map of proteins only identified at E14 and E16. Strong association between the proteins, which are associated with cardiac muscle contraction, as well as between the proteins involved in retinoid metabolic processes. B: Protein interaction map of proteins only identified at E19 and P1.

Supplementary Figure S4

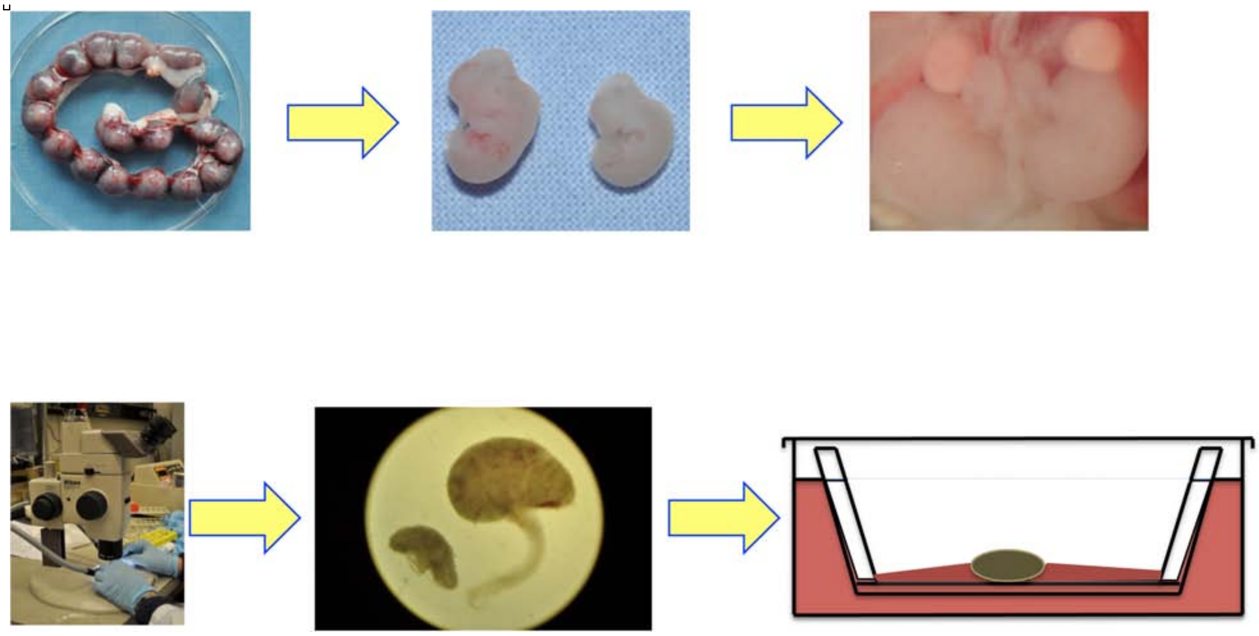

Supplemental Figure 4: Schematic view of the organ culture of the kidneys. The embryos were sacrificed at E13-14 and the kidneys were excised under a binocular. The kidneys were then isolated and plated on a transparent PET membrane with 0.4 µm pore size. The membrane was placed in one well of a 24 well plate containing 400 µl kidney culture medium.

Supplementary Figure S5

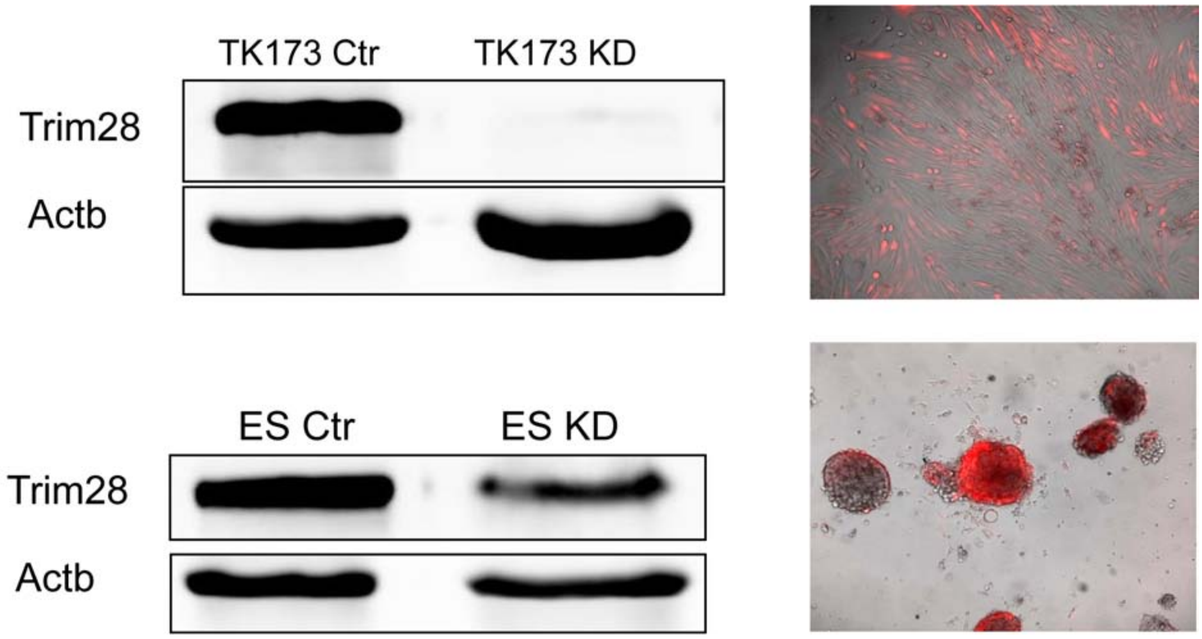

Supplemental Figure 5: Knockdown of Trim28 with psiRNA was tested on human kidney cells (TK173) and on murine embryonic stem cells. The knockdown was almost 100% in TK173 and > 50% in ESC. The transfection was monitored by microscopy, as the used plasmids contained a GFP insert (red).

Supplementary Figure S6

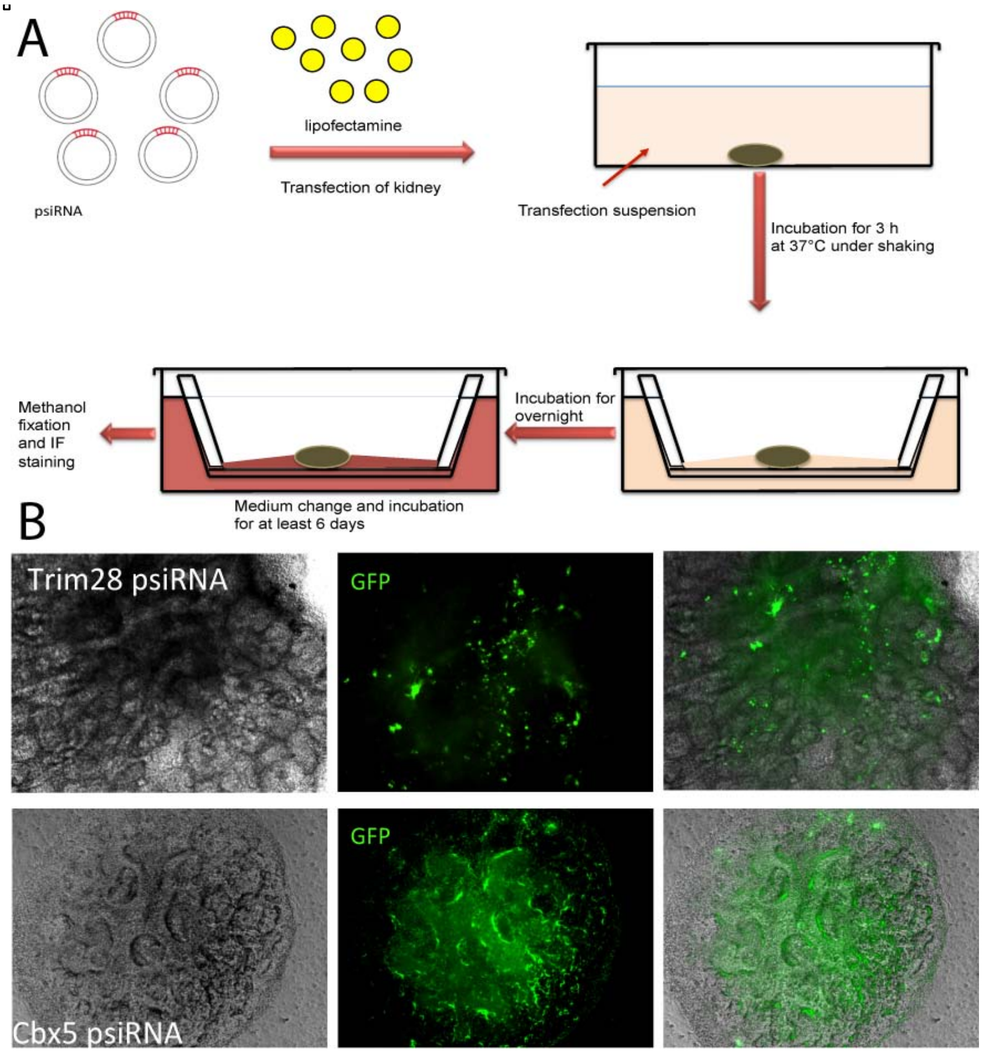

Supplemental Figure 6: A: Schematic protocol of the transfection of cultured embryonic kidneys with psiRNA. The plasmid inserted with shRNA was mixed with lipofectamine. The kidney was incubated for 3 h at 37 °C under shaking in the transfection suspension. Thereafter the transfection solution was removed and only a drop of the transfection solution was added on to the kidney. The rudiment on the PET membrane was placed in 24 well plates for attachment. The media was changed after 24 h and the kidney was cultured for up to 6 days. B: Monitoring the kidney transfection. Due to the GFP insert in the plasmid, the transfection of the kidney could be monitored by imaging. E14 kidneys were transfected either with Trim28 or Cbx5 psiRNA and cultured for additional 4 days. The transfection Efficiency was monitored by GFP expression in the kidney.

Supplementary Figure S7

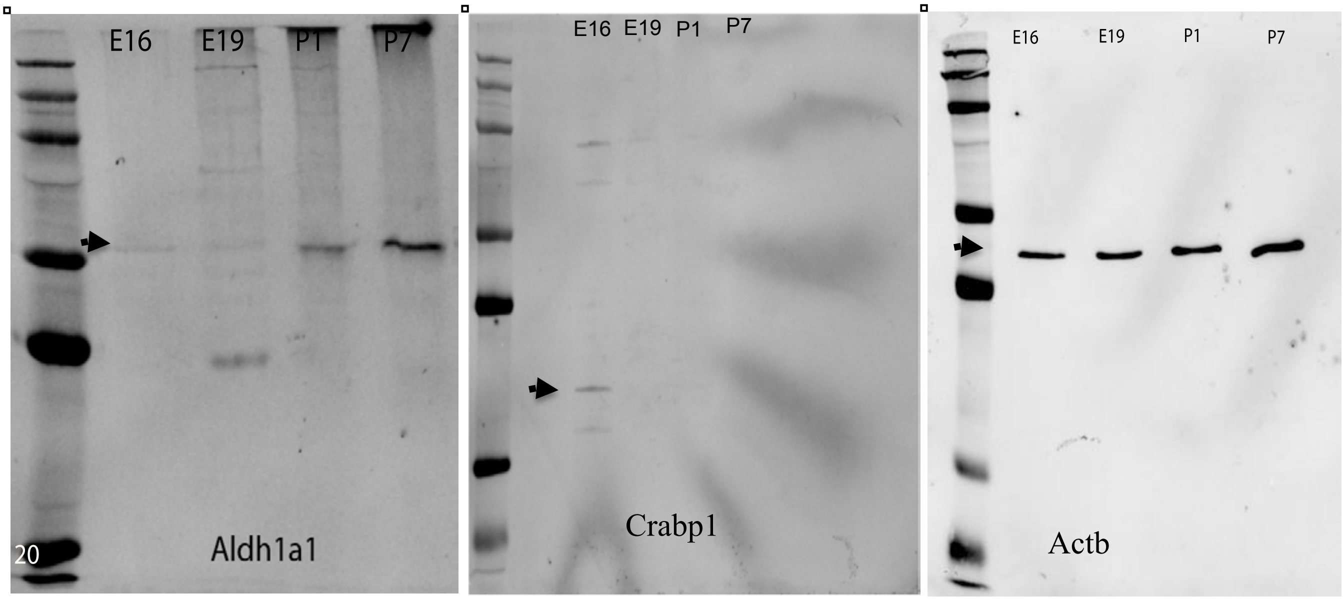

Supplementary Figure S7: Western blot analysis form embryonic kidney in different stages of development
Crude extract 60 µg were prepared from kidneys prepared from Embryos in day 16 and 19 of development and form new natal pubs and 7 days old rat.

Supplementary Figure S8

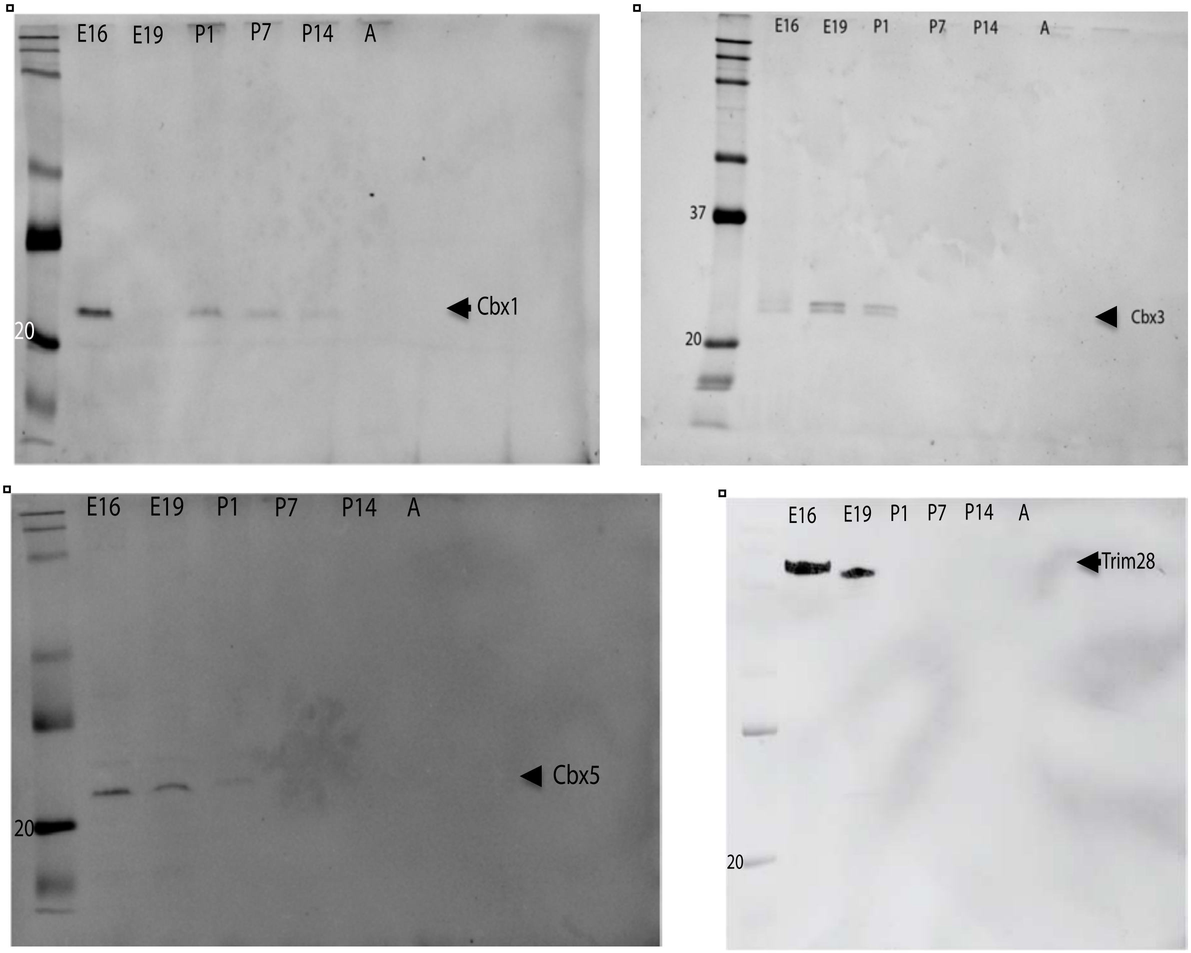

Supplementary Figure S8: Western blot analysis form embryonic kidney in different stages of development
Crude extract 60 µg were prepared from kidneys prepared from Embryos in day 16 and 19 of development and form new natal pubs and 7, 14 days old rat and adult animals.

Supplementary Figure S9

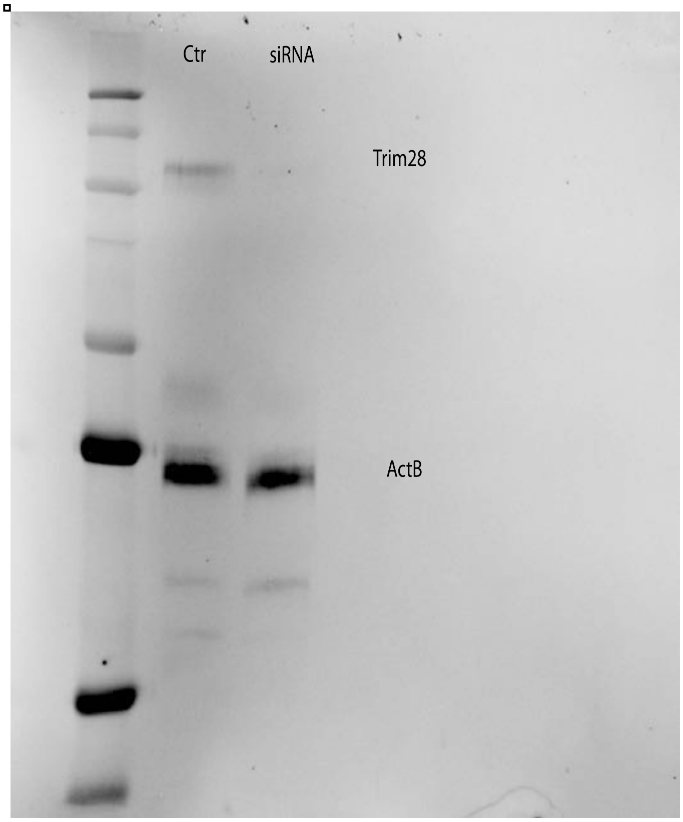

Supplementary Figure S9: Western blot analysis kidney for knockdown validation of targeted proteins.
The kidney rudiments were prepared form E13.5 and cultured overnight. Thereafter the cultured rudiments were treated with siRNA for the targeted protein or with lipofectamin as control. Crude extract then prepared from the cultured kidneys and used for Western blot.

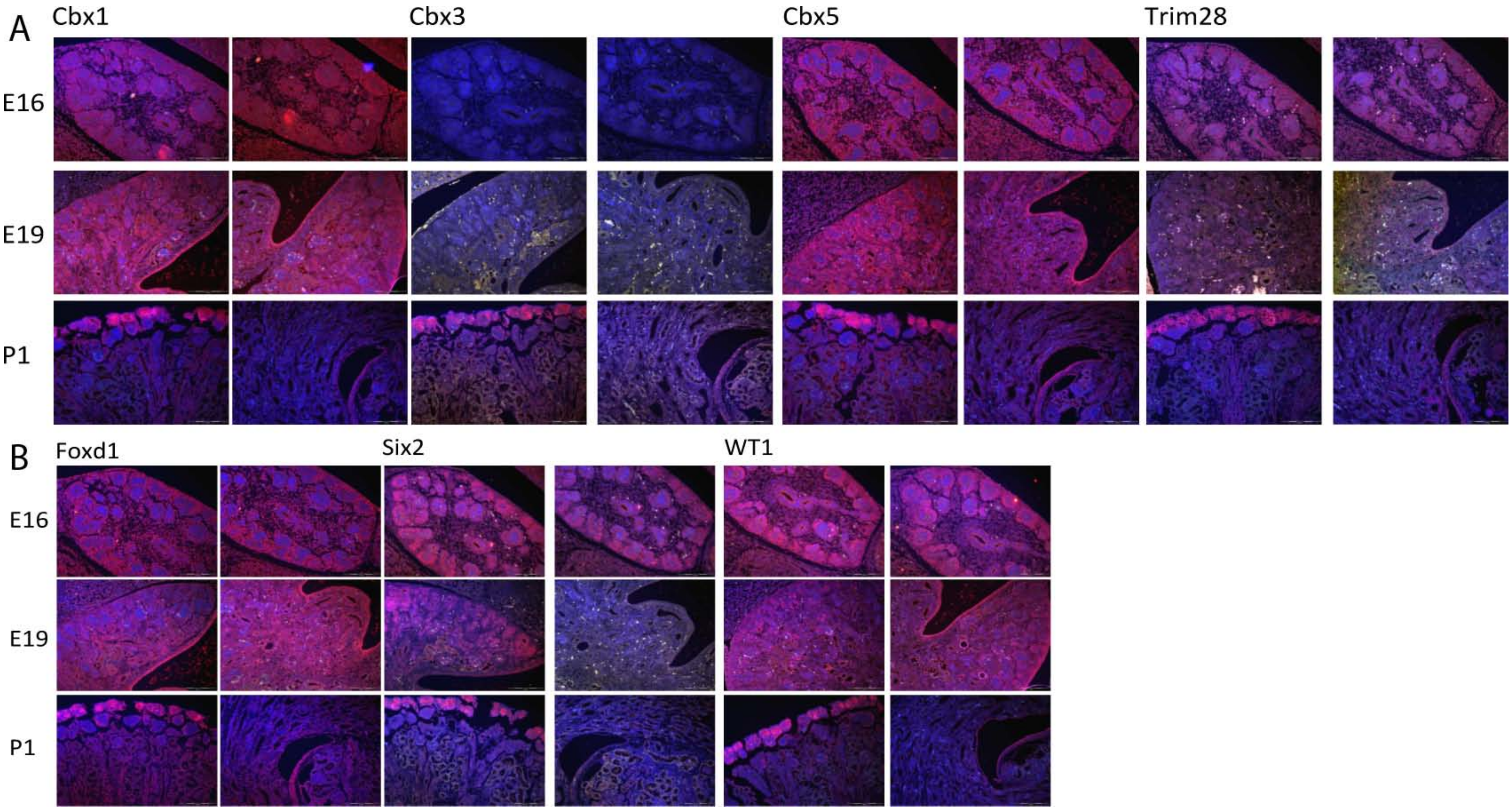

Supplemental Figure 10: Immunofluorescence staining of kidney section at different stage of development. A: Immunofluorescence staining with antibodies against Cbx1, 3, 5 and Trim28 (red) and DAPI (blue) for nucleus staining. The sections were prepared form E16, E19 and P1 kidneys. B: Immunofluorescence staining with antibodies against Foxd1, Six2 and Wt1 (red) and DAPI (blue) for nuclear staining.

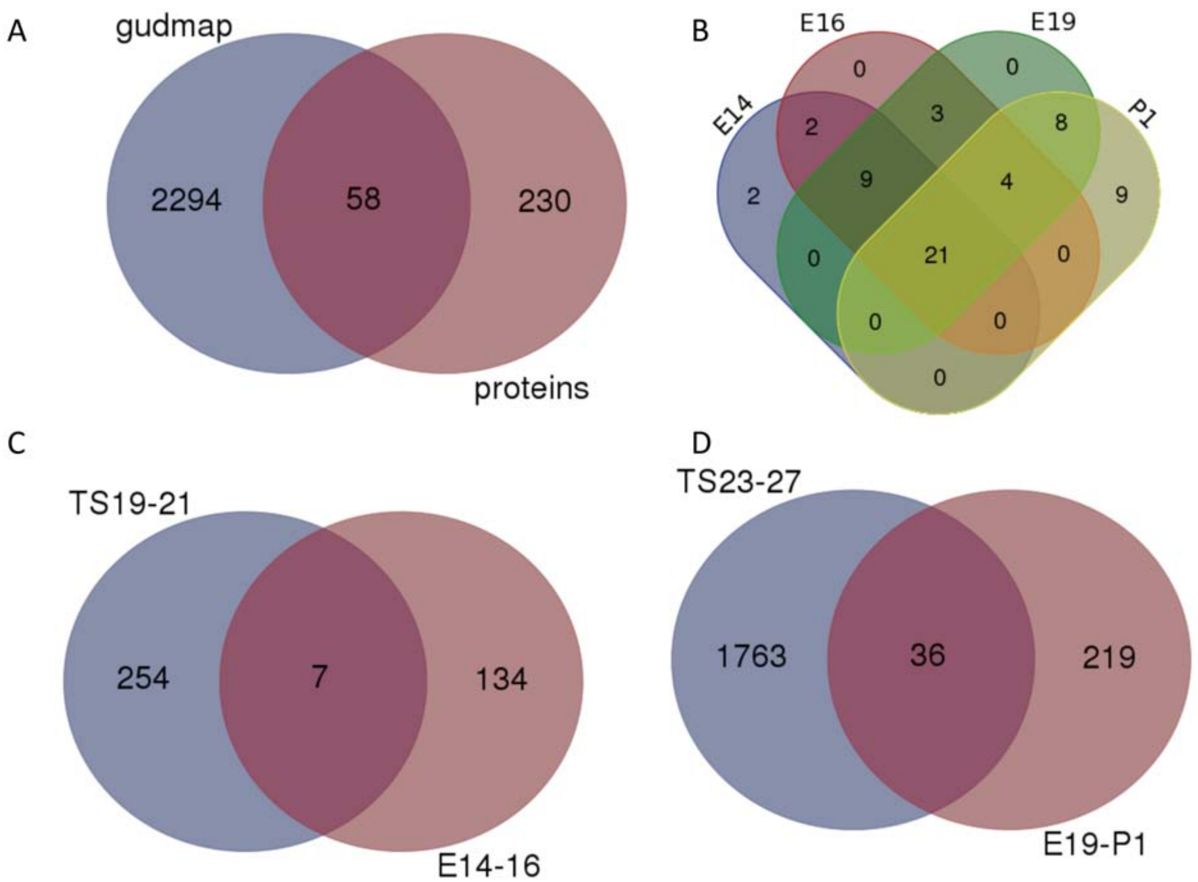

Supplemental Figure 11: A: In the GUDMAP database, all of the genes under the query „kidney" were selected, and compared to the proteins (288 non-redundant), identified in our study using the Bioinformatics and system biology software: (http://bioinformatics.psb.ugent.be/webtools/Venn/). 58 proteins overlapped with our results. B: The overlapping genes classified after the kidney development stage. C: Genes found in early developmental stages TS 19-21 were also analysed. Aldh1a2 Pgam1 Tubb5 Rbp1 Actc1 Lgals1 Eno1 were among the overlapping genes. D: at late stagesTS 23-27 (Overlapping genes: Strap Dld Gss Krt8 Fbp1 Vim Glud1 Aldoa Ldha Aldh1a3 Stmn1 Gatm Afp Cnn3 Pter Tardbp Capg Prdx1 Khsrp Prdx5 Psmd14 Tpi1 Zyx Dlst Uqcrc1 Aldh1a2 Ezr Aldh9a1 Acat2 Ttr Aldob Gsto1 Fabp3 Btf3 Lgals1 Ctsb).

Cell Culture

The human renal fibroblast cell line (TK 173) used in these experiments was derived from a normal human kidney biopsy and immortalized by transfection with the plasmid pSV3gpt which encodes the large T antigen from SV40[1]. The cells were maintained as described previously[2] The ESC R1 line was derived from the 129/Sv mouse line[3], and maintained as described.

(1) Müller, G. A.; Frank, J.; Rodemann, H. P.; Engler-Blum, G. Human renal fibroblast cell lines (tFKIF and tNKF) are new tools to investigate pathophysiologic mechanisms of renal interstitial fibrosis. *Exp. Nephrol.* **1995**, *3*, 127–133.

(2) Dihazi, H.; Dihazi, G. H.; Mueller, C.; Lahrichi, L.; Asif, A. R.; Bibi, A.; Eltoweissy, M.; Vasko, R.; Mueller, G. A. Proteomics characterization of cell model with renal fibrosis phenotype: osmotic stress as fibrosis triggering factor. *J. Proteomics* **2011**, *74*, 304–318.

(3) Nagy, A.; Rossant, J.; Nagy, R.; Abramow-Newerly, W.; Roder, J. C. Derivation of completely cell culture-derived mice from early-passage embryonic stem cells. *Proc. Natl. Acad. Sci. U. S. A.* **1993**, *90*, 8424–8428.
